# Spatiotemporal requirements of nuclear β-catenin define early sea urchin embryogenesis

**DOI:** 10.1371/journal.pbio.3002880

**Published:** 2024-11-12

**Authors:** Guy Lhomond, Michael Schubert, Jenifer Croce

**Affiliations:** Sorbonne Université, CNRS, Institut de la Mer de Villefranche (IMEV), Laboratoire de Biologie du Développement de Villefranche-sur-Mer (LBDV), Evolution of Intercellular Signaling in Development (EvoInSiDe), Villefranche-sur-Mer, France; Center for Genome Engineering, REPUBLIC OF KOREA

## Abstract

Establishment of the 3 primordial germ layers (ectoderm, endoderm, and mesoderm) during early animal development represents an essential prerequisite for the emergence of properly patterned embryos. β-catenin is an ancient protein that is known to play essential roles in this process. However, these roles have chiefly been established through inhibition of β-catenin translation or function at the time of fertilization. Comprehensive analyses reporting the totality of functions played by nuclear β-catenin during early embryogenesis of a given animal, i.e., at different developmental stages and in different germ layers, are thus still lacking. In this study, we used an inducible, conditional knockdown system in the sea urchin to characterize all possible requirements of β-catenin for germ layer establishment and patterning. By blocking β-catenin protein production starting at 7 different time points of early development, between fertilization and 12 h post fertilization, we established a clear correlation between the position of a germ layer along the primary embryonic axis (the animal-vegetal axis) and its dependence on nuclear β-catenin activity. For example, in the vegetal hemisphere, we determined that the 3 germ layers (skeletogenic mesoderm, non-skeletogenic mesoderm, and endoderm) require distinct and highly specific durations of β-catenin production for their respective specification, with the most vegetal germ layer, the skeletogenic mesoderm, requiring the shortest duration. Likewise, for the 2 animal territories (ectoderm and anterior neuroectoderm), we established that their restriction, along the animal-vegetal axis, relies on different durations of β-catenin production and that the longest duration is required for the most animal territory, the anterior neuroectoderm. Moreover, we found that 2 of the vegetal germ layers, the non-skeletogenic mesoderm and the endoderm, further require a prolonged period of nuclear β-catenin activity after their specification to maintain their respective germ layer identities through time. Finally, we determined that restriction of the anterior neuroectoderm territory depends on at least 2 nuclear β-catenin-dependent inputs and a nuclear β-catenin-independent mechanism. Taken together, this work is the first to comprehensively define the spatiotemporal requirements of β-catenin during the early embryogenesis of a single animal, the sea urchin *Paracentrotus lividus*, thereby providing new experimental evidence for a better understanding of the roles played by this evolutionary conserved protein during animal development.

## Abstract 2

Beta-catenin plays essential roles during the early embryogenesis of many animals, but these roles have been established mainly at the time of fertilization. This study uses an inducible knockdown system to determine the spatiotemporal roles played by nuclear b-catenin during early embryogenesis of sea urchin.

## Introduction

β-catenin is a pleiotropic protein that fulfills 2 main functions in animals [1,2]. At the cell membrane, it serves as a major component of the cell–cell adhesion machinery, acting as a mediator between cadherins and the actin cytoskeleton. In the cell nucleus, it acts as a transcriptional regulator, being a pivotal effector of the canonical Wnt signaling pathway. In this context, in the absence of an active Wnt signal, cytoplasmic β-catenin proteins are marked for degradation by the canonical Wnt signaling destruction complex, composed of Axin, APC (Adenomatous Polyposis Coli), GSK3β (Glycogen Synthase Kinase 3β), and CK1α (Casein Kinase 1α) [3,4]. Upon activation of the canonical Wnt pathway by extracellular binding of a Wnt ligand to a Frizzled receptor, the phosphoprotein Dishevelled is activated, binds to Axin, and thereby disrupts formation of the destruction complex. This disruption in turn stabilizes β-catenin proteins in the cytoplasm that subsequently translocate into the nucleus and bind to members of the TCF/LEF family of transcription factors, which ultimately mediate the transcriptional activation of Wnt signaling target genes [3,4].

Nuclear accumulation of β-catenin proteins has already been reported in a variety of different animal species, ranging from sponges to vertebrates [2,5–7]. In most animals, nuclear β-catenin is asymmetrically distributed along the primary embryonic axis, with a preferential accumulation in vegetal or posterior cells. Accordingly, nuclear β-catenin, and more generally the canonical Wnt pathway, acts as an important mediator of embryonic patterning along the primary body axes and as a key determinant of vegetal or posterior cell fates [8–10]. In some animals, nuclear β-catenin activity has also been reported to be context-specific. In the frog *Xenopus laevis*, for example, nuclear β-catenin acts during early embryogenesis to promote dorsal fates, while later in development it promotes ventrolateral fates [11]. In the ascidian *Ciona intestinalis*, a sustained ON-ON activity of nuclear β-catenin is required for endoderm specification, while an ON-OFF activity is sufficient for mesoderm development [12]. During mouse development, the concentration of nuclear β-catenin is directly correlated with developmental progression in both pre- and post-gastrulating embryos, with different tissue types requiring different levels of nuclear β-catenin [13]. Likewise, in the sea star *Patiria miniata*, nuclear β-catenin is involved, in a dose-dependent manner, in endomesoderm development [14]. Despite this knowledge, comprehensive analyses revealing the totality of roles played by nuclear β-catenin during early embryogenesis of a given animal are still lacking.

In sea urchins, nuclear accumulation of β-catenin has also been reported. In *Paracentrotus lividus*, fertilization has been shown to trigger a drastic burst of β-catenin mRNA translation [15], and, in *Lytechinus variegatus*, nuclear entry of endogenous β-catenin proteins has been described as starting at the late 16-cell stage (corresponding to late fourth cleavage) [6]. From this stage onward, nuclear β-catenin is accumulated specifically in the most vegetal cells of the *L*. *variegatus* embryo, i.e., in the micromeres and macromeres, which correspond to the progenitors of the 3 vegetal germ layers (skeletogenic mesoderm, non-skeletogenic mesoderm, and endoderm). Just before the onset of gastrulation, in *L*. *variegatus*, β-catenin nuclearization starts disappearing from micromere descendants, while the nuclear signal remains detectable in macromere descendants. Functional analyses carried out in a number of sea urchin species, including *P*. *lividus*, *L*. *variegatus*, and *Strongylocentrotus purpuratus*, have further led to the definition of the molecular mechanisms controlling the nuclear accumulation of β-catenin in the micromeres and macromeres. In the micromeres, for example, nuclear entry of β-catenin has been suggested to take place independently of Wnt-Frizzled interactions and to depend instead on the enrichment of Dishevelled proteins present in the vegetal cortex of the egg and inherited by the micromeres [6,16,17]. Likewise, accumulation of β-catenin in macromere nuclei has been suggested, from work carried out in *S*. *purpuratus*, to be independent of Wnt-Frizzled interactions and to depend instead on the early enrichment of Dishevelled proteins [16]. In *P*. *lividus*, however, we have previously established that the specific nuclear accumulation of β-catenin in the macromeres at the 32-cell stage relies on the interplay of the Wnt6 ligand and the Frizzled1/2/7 receptor [18].

**Fig 1:**
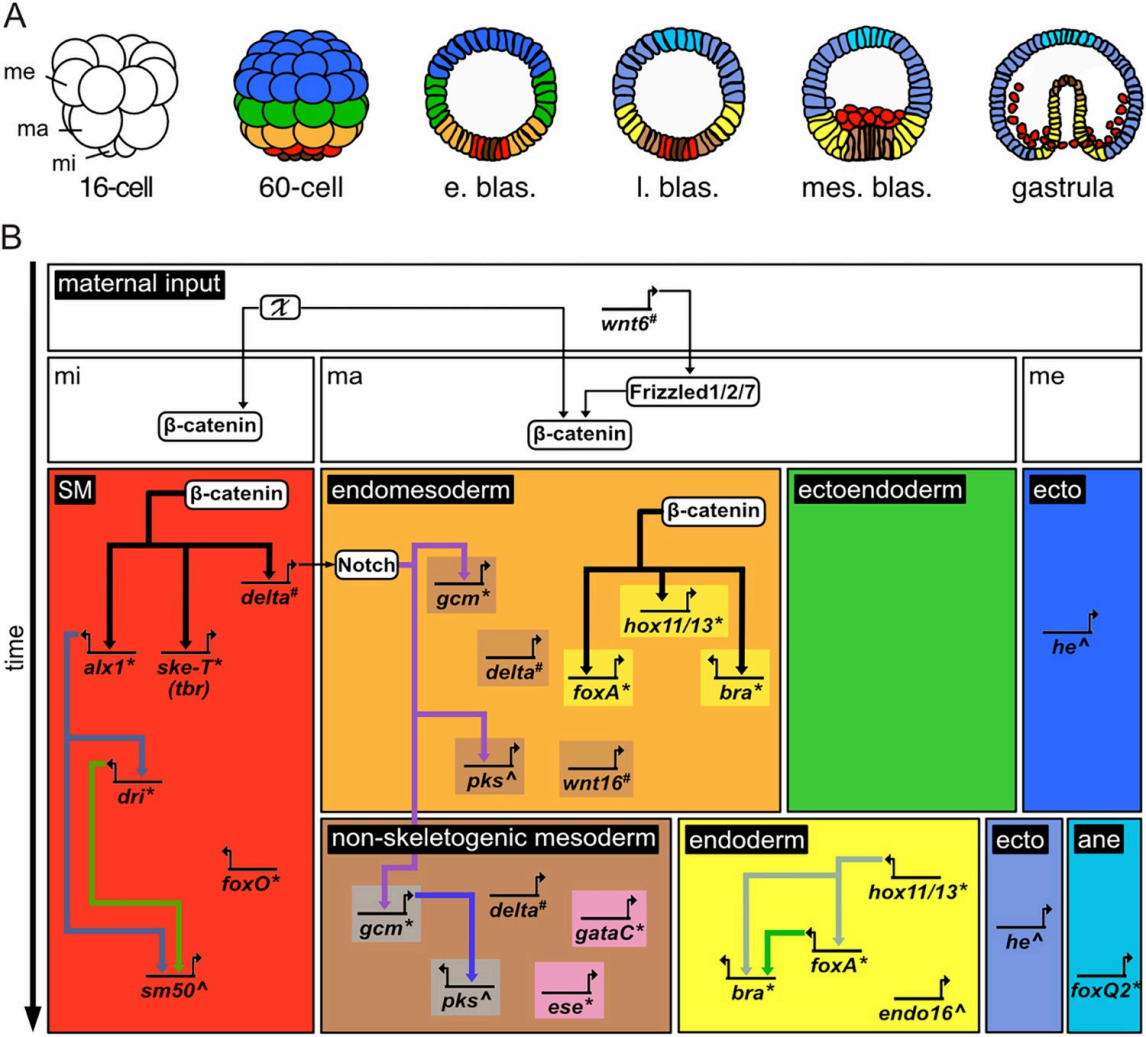
Simplified sea urchin GRN. (A) Schematics illustrating the germ layers of a sea urchin at 6 embryonic stages: 16-cell stage (16-cell), 60-cell stage (60-cell), early blastula stage (e. blas.), late blastula stage (l. blas.), mesenchyme blastula stage (mes. blas.), gastrula stage (gastrula). Embryonic domains are color-coded as follows: anterior neuroectoderm, cyan; ectoderm, intense and light blue; ectoendoderm, green; endoderm, yellow; endomesoderm, orange; non-skeletogenic mesoderm, light brown; skeletogenic mesoderm, red; small micromeres, dark brown; undetermined, white. Abbreviations: ma, macromeres; me, mesomeres; mi, micromeres. (B) Simplified sea urchin GRN based on data from *Paracentrotus lividus*, *Lytechinus variegatus*, and *Strongylocentrotus purpuratus* (e.g., [6,17,18,23–25]). In the GRN, each embryonic domain is highlighted by boxes using the same color-code as in (A), plus gray for the aboral (dorsal) non-skeletogenic mesoderm and pink for the oral (ventral) non-skeletogenic mesoderm. Genes are named based on their corresponding proteins and indicated as lines with bent arrows. Proteins are shown in white boxes. The GRN highlights the germ layer distribution of investigated genes and known regulatory relationships between them whether direct or indirect (arrows pointing to transcriptional activation). The GRN further illustrates reported activation mechanisms leading to β-catenin nuclearization and known nuclear β-catenin target genes. *, ^#^, and ^^^, respectively indicate genes encoding transcription factors, signaling system components, and differentiation marker genes. Developmental progression is from top to bottom. Abbreviations: ane, anterior neuroectoderm; ecto, ectoderm; ma, macromeres; me, mesomeres; mi, micromeres; SM, skeletogenic mesoderm; X, unknown activation mechanism.

In sea urchins, functional analyses targeting β-catenin and the canonical Wnt pathway have also already determined the main roles played by this intercellular signaling system during embryogenesis. These studies, performed on different sea urchin species (chiefly *P*. *lividus*, *L*. *variegatus*, and *S*. *purpuratus*), have established that the canonical Wnt pathway and nuclear β-catenin are required for the development of the 3 vegetal germ layers (skeletogenic mesoderm, non-skeletogenic mesoderm, and endoderm) [6,17,19,20] as well as for the restriction, along the animal-vegetal axis, of the 2 animal territories (ectoderm and anterior neuroectoderm) [21–23]. Thanks to the tremendous amount of work performed by the sea urchin community, developmental gene regulatory networks (GRNs) have been established for each of these germ layers [24,25]. These GRNs have revealed the identities of the transcription factors, signaling pathway components, and differentiation marker genes involved in the development of each of these germ layers, along with the regulatory relationships between them. Fig 1 illustrates a subset of this information, focusing on the embryonic domains and genes investigated in this study. However, in sea urchins, the developmental functions of nuclear β-catenin have so far only been studied by targeting the activity of the canonical Wnt pathway before or at the time of fertilization. Yet, during early sea urchin embryogenesis, β-catenin proteins accumulate in the descendants of the micromeres and the macromeres until at least the onset of gastrulation [6]. Moreover, the vegetal germ layers are gradually established during early sea urchin embryogenesis, with, for example, the skeletogenic mesoderm segregating first from an endomesoderm lineage that subsequently splits into endoderm and non-skeletogenic mesoderm, usually just before gastrulation [26–28]. Thus, these observations support the idea that nuclear β-catenin may act at different stages of sea urchin embryogenesis to control different developmental processes.

Here, to comprehensively determine the spatiotemporal roles of nuclear β-catenin during sea urchin early embryogenesis, we developed an inducible, conditional knockdown approach based on the combination of a morpholino antisense oligonucleotide and a photocleavable morpholino sense oligonucleotide [29]. Using this approach, we found that inhibition of β-catenin translation, starting before fertilization and at 6 different developmental stages post fertilization, distinctively impacts embryogenesis, and more specifically vegetal germ layer development and animal territory restriction. We established a clear correlation between the position of the germ layer along the animal-vegetal axis and its need for a longer production period of β-catenin proteins: the further from the vegetal pole, the longer β-catenin production is necessary. Due to the lack of antibodies recognizing endogenous sea urchin β-catenin proteins, we were unable, however, to assess the nuclear dynamics of β-catenin following induction of the knockdowns. Previous work in human and mouse cell lines [30,31] as well as in frog embryos [32] has shown that the half-life of endogenous β-catenin proteins is about 60 min. In the sea urchin *L*. *variegatus*, the half-life of GFP-tagged *X. laevis* β-catenin proteins has also been estimated at about 60 min in macromere descendant cells [17]. Yet, in *L*. *variegatus*, the half-life of nuclear β-catenin has been shown to depend on the embryonic cell type considered, with the half-life varying between about 15 min in mesomeres and 100 min in micromeres. Moreover, in mice and sea stars, it has previously been described that the regulatory functions of β-catenin proteins are dose-dependent [13,14] and that this dose-dependency varies between developmental stage, cell type, and even target gene. For all these reasons, we thus chose to present and interpret our results in the scope of developmental windows of β-catenin protein production rather than of its nuclear activity, even though it is the nuclear activity of β-catenin proteins that elicits molecular responses.

Taken together, this study defines the time course requirements of β-catenin production during the early embryogenesis of the sea urchin *P*. *lividus*. As such, it represents, to our knowledge, the first comprehensive description, in a single species, of the spatiotemporal functions of β-catenin during early development. The experimental evidence reported here thus provides a better understanding of the overall complexity of β-catenin actions, and more generally of the canonical Wnt signaling system, during animal development.

## Results

### Vegetal germ layer segregation during *Paracentrotus lividu*s embryogenesis

In the sea urchins *L*. *variegatus* and *S*. *purpuratus*, segregation of the 3 vegetal germ layers (skeletogenic mesoderm, non-skeletogenic mesoderm, and endoderm) has already been reported. These studies have shown that, while in both species the skeletogenic mesoderm segregates at fifth cleavage (32-cell stage) [33,34], the subsequent segregation of the endomesoderm, into non-skeletogenic mesoderm and endoderm, takes place at different stages and developmental times post fertilization. [26,28]. For *L*. *variegatus*, for example, segregation of the endomesoderm takes place before hatching, between 8 h post fertilization (hpf) and 9 hpf, whereas for *S*. *purpuratus* it happens after hatching, between 18 hpf and 21 hpf. In *P*. *lividus*, a thorough analysis of endomesoderm segregation is still lacking. However, given the importance of β-catenin for the development of the vegetal germ layers in sea urchins and other animals, we decided to investigate this event in this species as well. For this, double *in situ* hybridization assays were carried out using the 3 classical marker genes employed for the analysis of vegetal germ layer segregation in sea urchins: *delta*, *gcm*, and *foxA* [26,28]. We further investigated this process from 9 hpf (mid-blastula stage), i.e., when the expression of all three genes was detectable.

**Fig 2:**
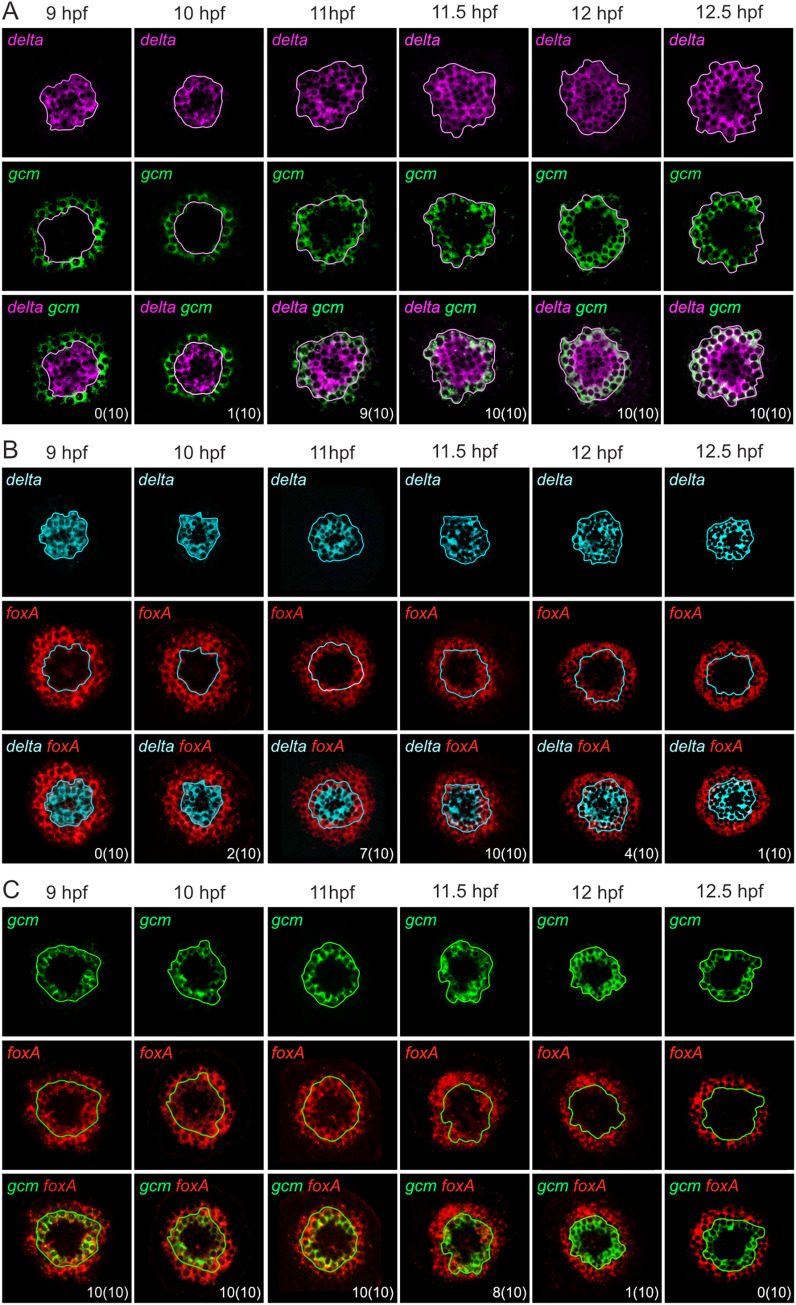
Vegetal germ layer segregation during *Paracentrotus lividu*s embryogenesis. (A–C) Double fluorescent *in situ* hybridization assays for: (A) *delta* (purple) plus *gcm* (green) mRNAs, (B) *delta* (blue) plus *foxA* (red) mRNAs, and (C) *gcm* (green) plus *foxA* (red) mRNAs. In (A–C), developmental stages are indicated as hpf and are as follows [37]: 9 hpf, mid-blastula stage; 10 hpf, late blastula stage; 11 hpf, hatched blastula stage; 11.5 hpf (11 h 30 min), swimming blastula stage; 12 hpf, swimming blastula stage; 12.5 hpf (12 h 30 min), swimming blastula stage. In (A–C), maximum intensity projections of confocal z-stacks for embryos in vegetal view. The first and second rows show the expression profiles of each indicated gene individually, and the third row shows a merge of the expression profiles of the 2 indicated genes. Numbers in the bottom right corner indicate the number of embryos with at least a couple of cells co-expressing the 2 analyzed genes, with the total number of embryos analyzed indicated in parentheses. In (A–C), color-coded lines, outlining the expression domain of one of the genes analyzed, are provided to facilitate visualization of co-expression.

At 9 hpf, *delta* was expressed exclusively in the precursors of the skeletogenic mesoderm, and thus in a ring of cells surrounding the vegetal pole (Fig 2A and 2B). The *delta*-expressing ring was about 2 cell layers thick and was limited, towards the animal pole, by a small ring of cells expressing *gcm* (1 cell layer thick) (Fig 2A) and a large ring of cells expressing *foxA* (2 cell layers thick) (Fig 2B). At 9 hpf, there was no co-expression of *delta* with either *gcm* or *foxA* (Fig 2A and 2B). In contrast, *gcm* and *foxA* were co-expressed in the *foxA*-positive cell layer closest to the *delta* ring (Fig 2C). These results support the idea that, at 9 hpf (mid-blastula stage), the skeletogenic mesoderm lineage is already segregated from the endomesoderm lineage (as highlighted by *delta* expression), while the endomesoderm lineage has not yet separated into non-skeletogenic mesoderm and endoderm (as shown by the co-expression of *foxA* and *gcm*).

Between 10 hpf and 11 hpf (late and hatched blastula stage, respectively), slight differences were observed in the expression patterns of *delta*, *gcm*, and *foxA* compared to the previous stage (Fig 2A–2C). For example, the expression territory of *delta* started to expand towards the animal pole, with cells progressively starting to co-express *delta* and *gcm* or *delta* and *foxA* (Fig 2A and 2B). By 11 hpf, *delta*, *gcm*, and *foxA* were thus co-expressed, in more than half of the scored embryos, in the cell layer adjacent to the skeletogenic mesoderm territory (Fig 2A–2C). This expansion of *delta* expression towards the future non-skeletogenic mesoderm territory is coherent with previous studies carried out in *L*. *variegatus* [26]. However, these results are inconsistent with the previously suggested segregation, by 11 hpf, of the endomesoderm lineage in *P*. *lividus* [18].

During the subsequent transition, i.e., from 11.5 hpf to 12.5 hpf (swimming blastula stages), the expression of *delta*, *foxA*, and *gcm* changed significantly (Fig 2A–2C). The *delta*-positive ring continued to expand towards the animal pole, completely covering, by 11.5 hpf, both the skeletogenic and non-skeletogenic mesoderm precursor cells in all scored embryos (Fig 2A and 2B). Likewise, expression of *gcm* expanded towards the animal pole, establishing a ring of 2 cell layers by 11.5 hpf (Fig 2B and 2C). Hence, from 11.5 hpf onward, *delta* and *gcm* were co-expressed, with the *gcm*-positive cells delineating the outer limit of the *delta*-positive ring (Fig 2A). During the 11.5 hpf to 12.5 hpf transition, expression of *foxA* also expanded towards the animal pole, but was further progressively lost from cells located inside the ring of expression (Fig 2B and 2C). Consequently, compared to earlier developmental stages, by 12.5 hpf, the *foxA* domain corresponded still to a ring that was 2 cell layers thick but was characterized by a larger diameter and a larger unlabeled zone in its center (Fig 2B and 2C). Moreover, during the 11.5 hpf to 12.5 hpf transition, the loss of *foxA* expression at the center of the ring was progressive, gradually leading to cells that became *gcm*-positive and *foxA*-negative (Fig 2C). During the swimming blastula stages, we thus observed a gradual commitment of endomesoderm cells to non-skeletogenic mesoderm or endoderm fates (Fig 2C). This progressive segregation of the endomesoderm lineage is consistent with results from single-cell transcriptome analyses in *L*. *variegatus* [35]. By 12.5 hpf, we therefore started to observe a majority of *P*. *lividus* embryos with no cell co-expressing *gcm* and *foxA* or *delta* and *foxA* (Fig 2B and 2C). These results hence indicate that by the swimming blastula stage, at around 12.5 hpf, all *P*. *lividus* embryos have completed segregation of the endomesoderm into endoderm and non-skeletogenic mesoderm and thus that at this stage the 3 vegetal germ layers are fully segregated.

### Patterns of β-catenin nuclearization during *Paracentrotus lividus* embryogenesis

Similar to endomesoderm segregation, the nuclear distribution of β-catenin proteins has previously been reported in the sea urchins *L*. *variegatus* and *S*. *purpuratus*, based on, respectively, endogenous and exogenous (fluorescently tagged) proteins [6,17,19]. Equivalent analyses in *P*. *lividus* are however still lacking. To address this, we generated a Venus-tagged form of *P*. *lividus* β-catenin, which we called β-cateninV and rendered regulatory inactive by site-directed mutagenesis in the TCF/LEF binding domain (S1 Fig). Based on previous reports, including from sea urchins [17,36], we expected the exogenous β-cateninV proteins to display a similar cellular distribution and stability as the endogenous β-catenin. Accordingly, at 7 hpf (very early blastula stage), we only observed nuclearization of β-cateninV proteins in the presumptive vegetal hemisphere of control embryos (S1 Fig). Similarly, we found that up-regulation of canonical Wnt pathway activity, upon lithium chloride (LiCl) treatment [6], resulted in β-cateninV nuclearization throughout most of the embryo, while down-regulation of canonical Wnt pathway activity, by overexpression of a truncated form of G-cadherin [6], completely abolished β-cateninV nuclearization (S1 Fig). As β-cateninV proteins are regulatory inactive, microinjection of different concentrations of mRNA encoding β-cateninV had no effect on sea urchin embryogenesis (at least until the 4-arm pluteus stage at 72 hpf) (S1 Fig). These experiments therefore validated the use of the β-cateninV construct to investigate the spatiotemporal dynamics of β-catenin nuclearization during *P*. *lividus* embryogenesis.

**Fig 3:**
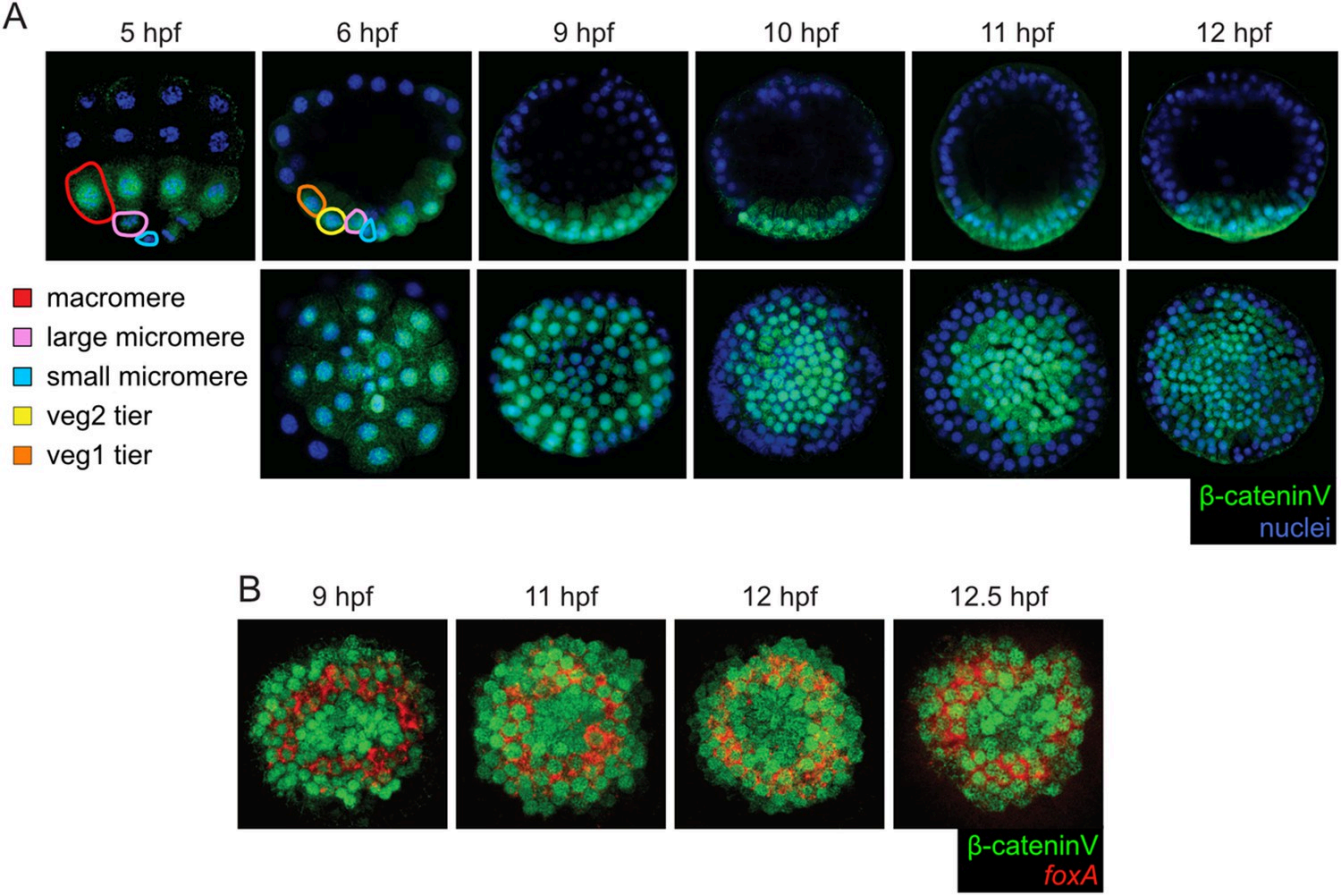
Nuclear localization of β-catenin during *Paracentrotus lividus* embryogenesis. (A, B) Maximum intensity projections of confocal z-stacks for embryos co-labeled with: (A) β-cateninV proteins (green) plus DNA (blue) and (B) β-cateninV proteins (green) plus *foxA* mRNAs (red). In (A, B), developmental stages are indicated as hpf and are as follows [37]: 5 hpf, 32-cell stage; 6 hpf, 60-cell stage; 9 hpf, mid-blastula stage; 10 hpf, late blastula stage; 11 hpf, hatched blastula stage; 12 hpf, swimming blastula stage; 12.5 hpf (12 h 30 min), swimming blastula stage. In (A), embryos in the top row are in lateral view with the animal pole up, and embryos in the bottom row are in vegetal view. At 5 hpf and 6 hpf, the red line outlines a macromere, the pink lines a large micromere, the light blue lines a small micromere, the orange line a veg1 cell, and the yellow line a veg2 cell. In (B), embryos are in vegetal view.

Using the β-cateninV construct, we then analyzed the distribution of nuclear β-catenin during *P*. *lividus* embryogenesis from fertilization to 12.5 hpf, the time point of complete segregation of the 3 vegetal germ layers (see Fig 2). Tag-specific immunochemistry revealed that nuclear accumulation of β-cateninV proteins was first detectable at 5 hpf (32-cell stage) (Fig 3A), which is coherent with the distribution of endogenous β-catenin proteins in *L*. *variegatus* [6]. At this stage, nuclear β-cateninV proteins were only present in the most vegetal cells: the 4 small and the 4 large micromeres and the 8 macromeres (Fig 3A). At 6 hpf (60-cell stage), nuclear β-cateninV proteins were still detectable only vegetally, in the descendants of the small and large micromeres and in those of the macromeres, which now compose the veg1 and veg2 cell tiers (Fig 3A). During the subsequent blastula stages, *P*. *lividus* embryos do no longer exhibit any specific landmark to distinguish the animal-vegetal axis or the different cell tiers [37]. Nevertheless, at 9 hpf (mid-blastula stage), we detected β-cateninV nuclearization only in a disc of about 80 cells that was located on one side of the embryo (Fig 3A), very likely corresponding to the vegetal side [6,17,19]. At 10 hpf and 11 hpf (late and hatched blastula stage, respectively), although the embryo underwent several rounds of cell divisions [37], nuclear β-cateninV proteins were still restricted to about 80 to 90 cells in the presumptive vegetal hemisphere (Fig 3A). Finally, at 12 hpf and 12.5 hpf (swimming blastula stages), β-cateninV nuclearization was still limited to one side of the embryo, but the total number of cells with nuclear β-cateninV increased to about 160 cells (Fig 3A and 3B). Of note, at 12 hpf and 12.5 hpf, we did not observe a marked clearance of nuclear β-cateninV proteins from presumptive skeletogenic mesoderm cells, as was previously reported for endogenous β-catenin proteins in *L*. *variegatus* [6]. However, we did observe such a clearance at 14 hpf (early mesenchyme blastula stage), when assessing the distribution of β-cateninV proteins in live embryos or by immunohistochemistry (S2 Fig). This difference between *L*. *variegatus* and *P*. *lividus* might be explained by: (1) a heterochrony between the 2 species; (2) a difference in defining developmental stages in the 2 species; or (3) a higher stability of β-cateninV proteins (assayed in *P*. *lividus*) compared to endogenous β-catenin proteins (assayed in *L*. *variegatus* [6]). Despite this difference, our analysis demonstrated that in *P*. *lividus*, as in *L*. *variegatus*, β-catenin proteins are restricted to the vegetal hemisphere of the embryo during cleavage and blastula stages.

Due to the absence of morphological landmarks in *P*. *lividus* blastulae, we next co-labeled embryos, from 9 hpf to 12.5 hpf, for *foxA* mRNAs and β-cateninV proteins to determine the precise localization of nuclear β-catenin proteins relative to the 3 vegetal germ layers (Fig 3B). At 9 hpf (mid-blastula stage), the *foxA*-positive ring was located at the center of the domain positive for nuclear β-cateninV proteins (Fig 3B). This result demonstrates that, at 9 hpf, β-cateninV proteins are nuclear: (1) in the small and large micromere descendants (i.e., in the cells located at the center of the *foxA*-positive ring) (which, respectively, correspond to the progenitors of a subset of the non-skeletogenic mesoderm cells and to the progenitors of the skeletogenic mesoderm lineage); and (2) in descendants of the veg1 and veg2 cell tiers (i.e., in cells positive for *foxA* and *gcm*, in cells positive only for *foxA*, and in cells located around the *foxA* domain) (which together correspond to the progenitors of both the endoderm and non-skeletogenic mesoderm lineages). At 11 hpf (hatched blastula stage), the *foxA*-positive ring was still nested within the nuclear β-cateninV-positive domain, while at 12 hpf and 12.5 hpf (swimming blastula stages), *foxA* mRNAs were found at the edge of the nuclear β-cateninV domain (Fig 3B). Between 11 hpf and 12 hpf, the domain of nuclear β-cateninV expanded (Fig 3A), and *foxA* expression shifted from cells in the vicinity of the micromere descendants to cells outside of the initial *foxA*-positive ring (see Fig 2B and 2C). These observations indicate that, by 12 hpf and 12.5 hpf, nuclear β-cateninV proteins are associated not only with the descendants of the small and large micromeres (i.e., the *delta*-positive cells), but also with the newly-segregated endoderm (i.e., the *foxA*-positive cells) and non-skeletogenic mesoderm (i.e., the *delta*- plus *gcm*-positive cells) lineages. These results further suggest that, although, at 9 hpf and 11 hpf (mid- and hatched blastula stage, respectively), the cells positive for nuclear β-cateninV located outside the *foxA*-positive ring are not committed to the endoderm lineage, they are already destined to become endoderm. In sum, we demonstrate that, throughout *P*. *lividus* blastula stages, β-catenin nuclearization is uniquely associated with cells destined to give rise to one of the 3 vegetal germ layers: the skeletogenic mesoderm, the non-skeletogenic mesoderm, and the endoderm.

### Methodology and validation of an inducible, conditional β-catenin knockdown approach

**Fig 4:**
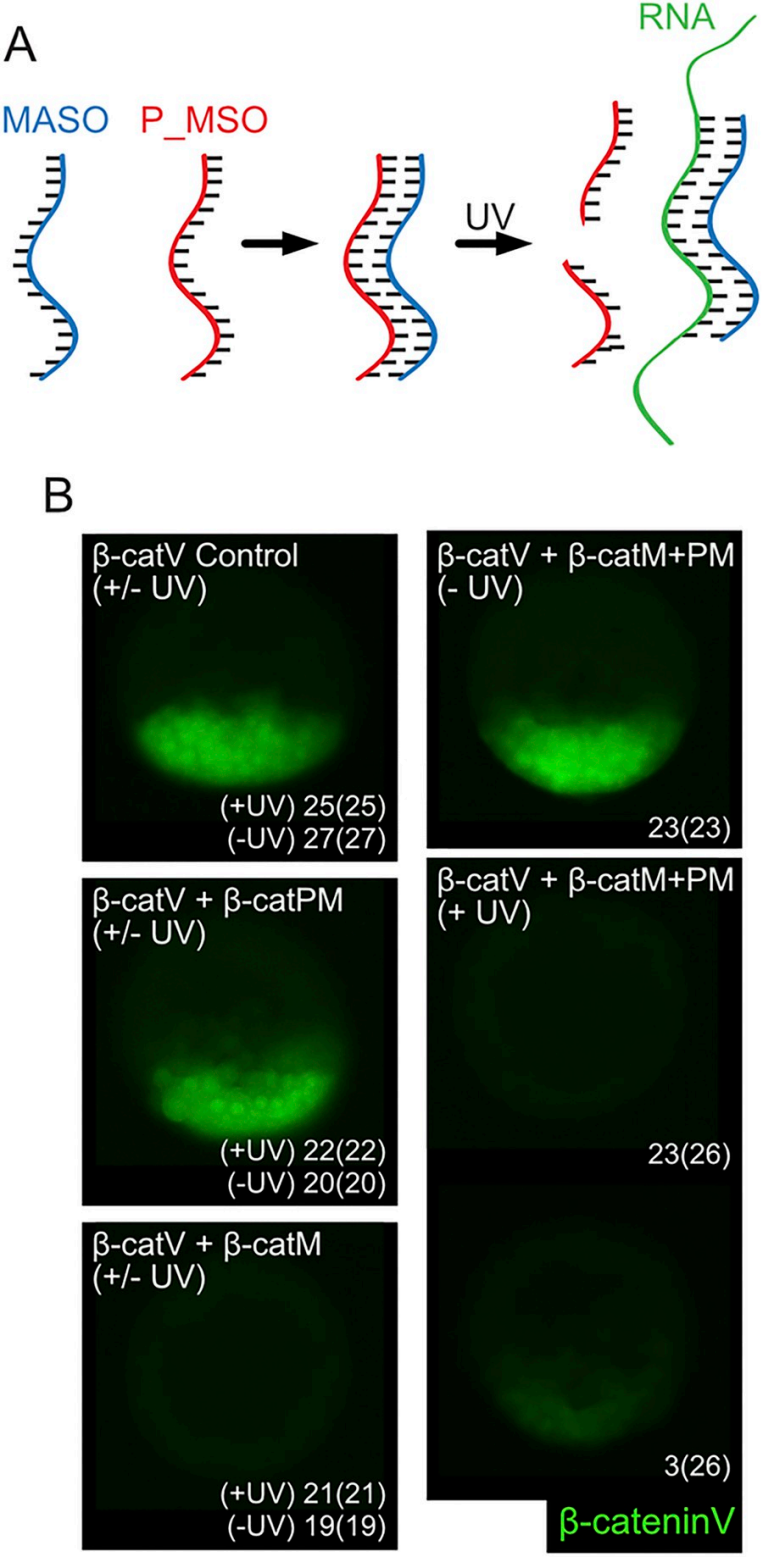
Methodology and validation of an inducible, conditional β-catenin knockdown system. (A) Schematic representation of an inducible, conditional gene knockdown approach developed based on the use of classical morpholino antisense oligonucleotides (MASO) and photocleavable morpholino sense oligonucleotides (P_MSO). The 2 morpholino oligonucleotides are complementary and bind to each other, thereby preventing the association of the MASO with its target mRNA. Upon UV light exposure (365 nm), P_MSO is cleaved, hence releasing the MASO, which in turn binds to its target mRNA, blocks its translation, and induces a knockdown phenotype. (B) *In vivo* validation of the developed inducible knockdown approach using the production of β-cateninV proteins (green) as a readout. Embryos were obtained from oocytes microinjected with β-cateninV mRNAs alone (control), with β-cateninV mRNAs plus β-catenin MASO (β-cateninM, β-catM), with β-cateninV mRNAs plus β-catenin P_MSO (β-cateninPM, β-catPM), or with β-cateninV mRNAs plus a mix of β-cateninM and β-cateninPM (β-catM+PM). Following microinjection, but before fertilization, oocytes were either exposed (+UV) or not exposed (-UV) to UV light. All embryos were then recorded live at 9 hpf (mid-blastula stage) [37] and are shown in lateral view, with the animal pole up. Numbers in the bottom right corner indicate counts of shown phenotypes with numbers in parentheses indicating the total number of scored embryos from 2 biological replicates.

To survey the spatiotemporal requirements of nuclear β-catenin during *P*. *lividus* embryogenesis, we next set up an inducible, conditional knockdown approach, allowing the blockage of β-catenin translation starting at any desired time during development [29]. The system uses a classical morpholino antisense oligonucleotide directed against *P*. *lividus* β-catenin (β-cateninM or β-catM) and a ultraviolet (UV)-photocleavable morpholino sense oligonucleotide complementary to β-cateninM (β-cateninPM or β-catPM) (Fig 4A). To validate the methodology, we first took advantage of the presence of the β-cateninM target sequence at the 5′ end of the β-cateninV construct (S1 Fig). mRNAs encoding β-cateninV proteins were thus microinjected into *P*. *lividus* oocytes either alone (control condition) or together with different morpholino oligonucleotide combinations, and the injected oocytes were then exposed (or not) to UV light before fertilization.

In control embryos, as in embryos microinjected with β-cateninPM, whether or not exposed to UV light, nuclear β-cateninV proteins were detectable, at 9 hpf (mid-blastula stage), in the presumptive vegetal pole region of all scored embryos (100%, 52 of 52 scored embryos = 52/52 for controls, 42/42 for β-cateninPM embryos) (Fig 4B). UV light exposure therefore did not affect embryogenesis or the distribution of nuclear β-cateninV proteins. Likewise, the presence of β-cateninPM did not induce any embryonic malformations or alterations of nuclear β-cateninV distribution. In contrast, in embryos microinjected with β-cateninV mRNA plus β-cateninM, whether exposed or not to UV light, no β-cateninV proteins were detectable at 9 hpf (mid-blastula stage) in any scored embryos (100%, 40/40) (Fig 4B). These results demonstrate the specificity of β-cateninM for its target sequence and that exposure to UV light does not affect this specificity. Finally, when embryos were microinjected with β-cateninV mRNAs along with β-cateninM plus β-cateninPM and were not exposed to UV light, we obtained embryos identical to those in the controls, with 100% of scored embryos (23/23) showing nuclear β-cateninV proteins in the presumptive vegetal region (Fig 4B). In contrast, in embryos microinjected with β-cateninV mRNAs and β-cateninM plus β-cateninPM and exposed to UV light, 88.5% of the scored embryos (23/26) were completely devoid of β-cateninV nuclearization, and the remaining 11.5% (3/26) were characterized by severely reduced levels of nuclear β-cateninV (Fig 4B). Taken together, these results establish the effective sequestration of β-cateninM by β-cateninPM in the absence of UV exposure and show that UV exposure is sufficient to release β-cateninM and to block translation of β-catenin mRNAs.

### Induced blockage of β-catenin translation before fertilization corroborates previous reports on β-catenin activity disruption before or at fertilization

**Fig 5:**
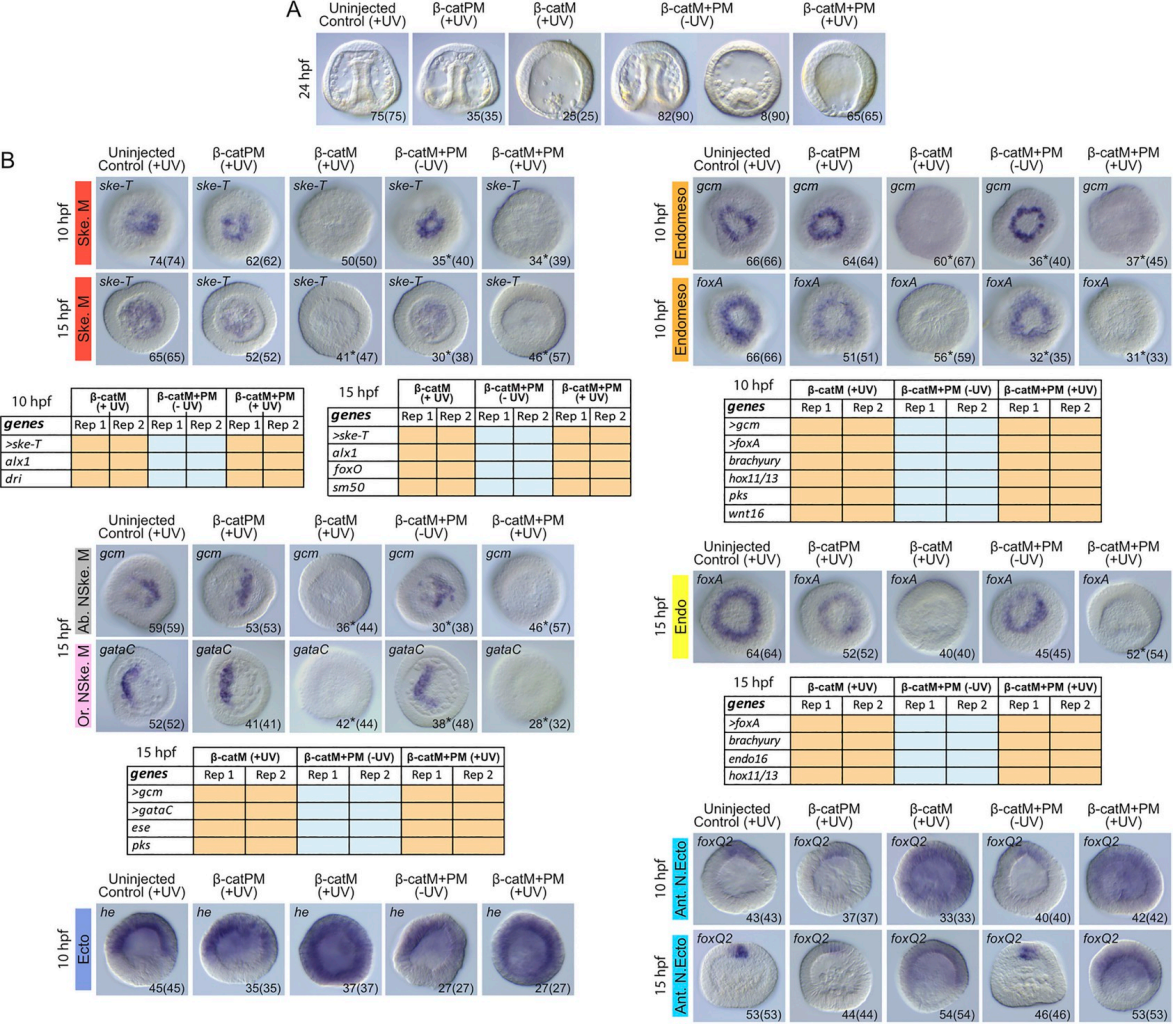
Developmental and molecular validation of the inducible β-catenin knockdown approach by blocking β-catenin translation before fertilization. (A) Morphological phenotypes observed at 24 hpf (late gastrula stage) in: uninjected control embryos (uninjected control), embryos microinjected with β-cateninPM (β-catPM) alone, with β-cateninM (β-catM) alone or with a mix of β-cateninM+β-cateninPM (β-catM+PM), which were all either irradiated (+UV) or not (-UV) before fertilization. All embryos are shown in lateral view, with the animal pole up. (B) *In situ* hybridization assays and qPCR expression matrices for markers of the skeletogenic mesoderm (Ske. M), the endomesoderm (Endomeso), the oral (ventral) non-skeletogenic mesoderm (Or. NSke. M), the aboral (dorsal) non-skeletogenic mesoderm (Ab. NSke. M), the endoderm (Endo), the ectoderm (Ecto), and the anterior neuroectoderm (Ant. N.Ecto). The color-code used for each germ layer is as in Fig 1. *In situ* hybridization and qPCR assays were carried out at 10 hpf (late blastula stage) and/or 15 hpf (mesenchyme blastula stage) [37], on embryos from the same microinjection and experimental setup as in (A). For the *in situ* hybridization, all embryos are shown in vegetal view, except for *he* and *foxQ2*, which are in lateral view. In (A, B), numbers in the bottom right corner indicate counts of shown phenotypes and *in situ* hybridization patterns, and the numbers in parentheses outline the total number of scored embryos. In (B), * indicates weak signals in the remaining scored embryos. In (B), the qPCR analyses are shown as tables and were carried out in 2 independent biological replicates (Rep1 and Rep2) on the genes analyzed by *in situ* hybridization (marked by >) as well as on additional marker genes. In the tables, orange backgrounds indicate down-regulation by more than 2-fold compared to uninjected, irradiated control embryos, while blue backgrounds highlight unaltered expression compared to controls. Related raw data and quantification methods are available in S1 Data and fold change values are further provided in S2 Data.

To further validate our inducible, conditional β-catenin knockdown approach *in vivo*, we next assessed the effects of a release of β-cateninM by UV treatment before fertilization and compared our results with those of previous reports on the down-regulation of canonical Wnt pathway activity before or at fertilization [6,17,19–23]. For this analysis, we included, as negative controls, uninjected oocytes and oocytes microinjected with β-cateninPM, both exposed to UV light, and, as positive controls, oocytes microinjected with β-cateninM and exposed to UV light. These controls were compared to oocytes microinjected with β-cateninM+β-cateninPM and exposed or not to UV light. The obtained phenotypes were analyzed (1) by assessing embryo morphology at 24 hpf (late gastrula stage) (Fig 5A); and (2) by *in situ* hybridization and quantitative RT-PCR (qPCR) assays (Fig 5B).

To investigate embryo morphology, we chose the late gastrula stage (24 hpf), a stage at which the germ layers have segregated and have formed easily recognizable morphological structures, such as, within the bastocoel, an elongated digestive tube, well-organized skeletogenic mesoderm cells, and scattered non-skeletogenic mesoderm cells, and, at the animal pole, a slightly inflated anterior neuroectoderm. For both negative controls (uninjected embryos and embryos microinjected with β-cateninPM), 100% of the scored embryos (75/75 and 35/35, respectively) developed into normal late gastrulae, with all expected morphological characteristics (Fig 5A). For the positive β-cateninM controls, 100% of the embryos (25/25) developed instead into animalized dauerblastulae. These embryos had no digestive tube in the blastocoel, and they exhibited an extended thick epithelium (Fig 5A), which is a phenotype consistent with a down-regulation of canonical Wnt signaling activity before or at fertilization [6,17,19,20]. Of the β-cateninM+β-cateninPM-injected embryos not exposed to UV light, 91% (82/90) developed into normal late gastrulae and 9% (8/90) had a delayed gastrula phenotype (Fig 5A). The delayed gastrula phenotype was characterized by the presence, in the blastocoel, of a small, developing digestive tube along with some mesodermal cells. In contrast, UV exposure of β-cateninM+β-cateninPM-injected oocytes resulted in 100% of animalized embryos (65/65) (Fig 5A). This morphological analysis thereby confirms the efficiency of our inducible β-catenin knockdown approach.

Gene expression surveys were subsequently carried out on embryos collected at 10 hpf (late blastula stage) and 15 hpf (onset of gastrulation, mesenchyme blastula stage) (Fig 5B), i.e., before and after the complete segregation of the 3 vegetal germ layers (for the rationale of the experimental setup, see Materials and methods). While for the vegetal cell lineages, we used both *in situ* hybridization and qPCR analyses, for the animal cell lineages, the analyses were limited to *in situ* hybridization assays. Moreover, for the vegetal lineages, we used the following marker genes: (1) 5 skeletogenic mesoderm markers, the transcription factors *ske-T* (*tbr*) and *alx1*, both expressed in this lineage at 10 hpf and 15 hpf [38–40], the transcription factors *deadringer (dri)* and *foxO*, respectively present in this lineage at 10 hpf and 15 hpf [41,42], and the differentiation gene *sm50*, restricted to this lineage at 15 hpf [43,44]; (2) 6 endomesodermal markers, the endoderm-related transcription factors *foxA*, *brachyury* (*bra*), and *hox11/13*, and the non-skeletogenic mesoderm-related transcription factor *gcm*, differentiation gene *pks*, and signaling molecule *wnt16*, all expressed in this lineage at 10 hpf [26,28,45–47]; (3) 2 oral (ventral) non-skeletogenic mesoderm markers, the transcription factors *gataC* and *ese*, both limited to this lineage at 15 hpf [48,49]; (4) 2 aboral (dorsal) non-skeletogenic mesoderm markers, the transcription factor *gcm* and the differentiation gene *pks*, both restricted to this lineage at 15 hpf [26,49]; and (5) 4 endoderm markers, the transcription factors *foxA*, *bra*, and *hox11/13* and the secreted glycoprotein *endo16*, all expressed in this lineage at 15 hpf [50–53]. For the animal lineages, we used one ectoderm marker, the metalloprotease *hatching enzyme* (*he*), expressed in this lineage at 10 hpf [21], and one anterior neuroectoderm marker, the transcription factor *foxQ2*, restricted to this territory at both 10 hpf and 15 hpf [54].

With regards to the vegetal lineages, our *in situ* hybridization and qPCR analyses revealed normal, unaltered expression of all markers, in both negative controls and embryos microinjected with β-cateninM+β-cateninPM and not exposed to UV light (Fig 5B). In contrast, when β-catenin translation was blocked before fertilization, either by microinjection of β-cateninM or by the release of β-cateninM from β-cateninPM by UV exposure, we observed a severe disruption of expression of all vegetal lineage markers, both by *in situ* hybridization and qPCR (Fig 5B). These results are consistent with previously reported defects induced by the down-regulation of canonical Wnt signaling activity before or at fertilization [6,17,19,20], and thus further corroborate the validity of our inducible β-catenin knockdown approach.

For the animal lineages, our marker gene survey chiefly yielded similar results. Embryos from both negative controls and from β-cateninM+β-cateninPM-injected oocytes not exposed to UV light were characterized by normal, unaltered expression of the ectoderm and anterior neuroectoderm markers (Fig 5B). In contrast, when β-catenin translation was blocked before fertilization, by injection of β-cateninM or of β-cateninM+β-cateninPM followed by UV light exposure, expression of the marker genes, at 10 hpf (late blastula stage), was significantly expanded towards the presumptive vegetal pole (Fig 5B). This result is again consistent with a down-regulation of canonical Wnt signaling activity before or at fertilization [21–23]. However, at 15 hpf (mesenchyme blastula stage), expression of the anterior neuroectoderm marker *foxQ2* was more restricted along the animal-vegetal axis than at 10 hpf, and this in all scored embryos (54/54 for β-cateninM embryos and 53/53 for β-cateninM+β-cateninPM+UV embryos) (Fig 5B). This suggests that restriction of the anterior neuroectodermal territory during *P*. *lividus* embryogenesis is not exclusively regulated by β-catenin nuclearization, with a nuclear β-catenin-independent mechanism being required at least during later steps of this process. Taken together, these experiments demonstrate the reliability of our inducible β-catenin knockdown approach, validating its use for the investigation of the spatiotemporal functions of β-catenin during *P*. *lividus* embryogenesis. Moreover, our results are indicative of a nuclear β-catenin-independent mechanism involved in restricting the anterior neuroectoderm territory along the animal-vegetal axis.

### Morphological defects associated with time point-specific blockage of β-catenin translation during *Paracentrotus lividus* embryogenesis

**Fig 6:**
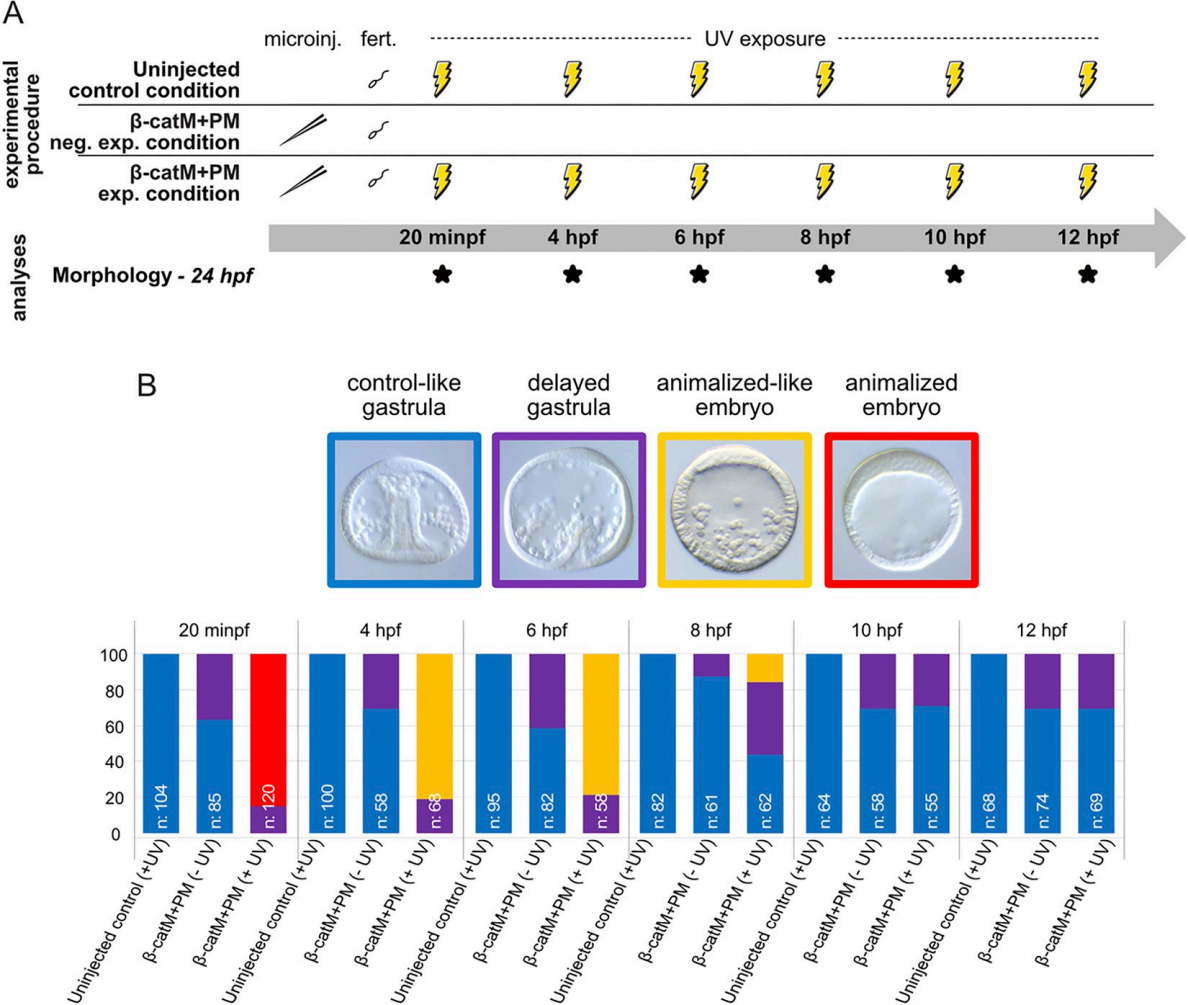
Morphological phenotypes associated with blocking β-catenin translation starting at different time points after fertilization. (A) Schematic representation of the experimental setup. Upon microinjection (or not) and fertilization, 6 developmental stages, indicated as minutes post fertilization (minpf) or hpf, were targeted for UV treatments and are as follows: 20 minpf, fertilized egg; 4 hpf, 16-cell stage; 6 hpf, 60-cell stage; 8 hpf, early blastula stage; 10 hpf, late blastula stage; 12 hpf, swimming blastula stage [37]. For all time points, embryo morphology was assessed at 24 hpf (late gastrula stage), as indicated by *. Abbreviations: exp. condition, experimental condition; fert., fertilization; microinj., microinjection; neg. exp. condition, negative experimental condition. (B) Quantification of the morphological phenotypes obtained at 24 hpf for uninjected, irradiated control embryos: uninjected control (+UV); β-cateninM+β-cateninPM-injected and not irradiated embryos: β-catM+PM (-UV); β-cateninM+β-cateninPM-injected and irradiated embryos: β-catM+PM (+UV). The top row illustrates the 4 different observed phenotypes named as indicated and presented with embryos in lateral view, with the animal pole up. The bottom graph highlights the quantification of each of the observed phenotypes, per experimental condition. This quantification is displayed using the same color-code as in the top row and is based on 3 biological replicates. The total number (n:) of embryos scored for each experimental condition is indicated on each histogram. Corresponding raw counts and quantification method are included in S3 Data.

Using our inducible, conditional knockdown approach, we first blocked β-catenin translation starting at different time points following fertilization (for the rationale of the experimental setup, see Materials and methods) and scored the morphology of the resulting embryos at 24 hpf (late gastrula stage) (Fig 6). The experimental setup included, as controls, uninjected embryos exposed to UV light at the selected time points and embryos injected with β-cateninM+β-cateninPM and not exposed to UV light, which were both compared to embryos injected with β-cateninM+β-cateninPM and exposed to UV light at the selected time points (Fig 6A). At 24 hpf, we found that 100% of the uninjected, irradiated control embryos (514/514) developed into normal late gastrulae, and this no matter when they were exposed to UV light (Fig 6B). By comparison, we observed that between 59% (48/82) (6 hpf) and 87% (53/61) (8 hpf) of the unexposed β-cateninM+β-cateninPM embryos also developed into normal late gastrulae, with the remaining 41% (34/82) (6 hpf) and 13% (8/61) (8 hpf) developing into delayed gastrulae (Fig 6B). Of note, we interpret the variability of the scores obtained for the unexposed β-cateninM+β-cateninPM embryos as fluctuations in the quality of oocyte batches used for microinjection or else as the result of faulty or incomplete microinjections.

When blocking β-catenin translation starting at 20 min post fertilization (minpf), we obtained 85% (102/120) of animalized embryos, with the remaining 15% (18/120) forming delayed gastrulae (Fig 6B). These percentages were similar to those obtained when β-catenin translation was blocked before fertilization (Fig 5A). In addition, from an anatomical point of view, no significant difference was detected between embryos that developed from oocytes irradiated before fertilization and embryos irradiated 20 minpf (compare Figs 5A and 6B). These results thus suggest that blocking β-catenin translation before fertilization or starting 20 minpf does not differently impact vegetal germ layer development and animal territory restriction in the *P*. *lividus* embryo, at least from a morphological point of view.

Subsequently, UV exposure of β-cateninM+β-cateninPM-injected embryos at 4 hpf and 6 hpf (16- and 60-cell stage, respectively) resulted chiefly in 2 phenotypes: an animalized-like phenotype in a majority of scored embryos (81% (55/68) for UV exposure at 4 hpf and 79% (46/58) for UV exposure at 6 hpf) and a delayed gastrula phenotype in the remaining embryos (19% (13/68) for UV exposure at 4 hpf and 21% (12/58) for UV exposure at 6 hpf) (Fig 6B). The majority of β-cateninM+β-cateninPM embryos exposed to UV light at 4 hpf and 6 hpf developed indeed into dauerblastulae, similar to those obtained after UV light exposure of β-cateninM+β-cateninPM embryos before fertilization or 20 minpf, but UV exposure at 4 hpf and 6 hpf resulted in a higher number of cells in the blastocoel and a reduced thickening of the epithelium along the animal-vegetal axis (Fig 6B). These results suggest that disruption of β-catenin protein production at either 4 hpf or 6 hpf thus still affects vegetal germ layer development and animal territory restriction.

Releasing β-cateninM at 8 hpf (early blastula stage) yielded 16% (10/62) of animalized-like embryos, 40% (25/62) of delayed gastrulae, and 44% (27/62) of normal late gastrulae (Fig 6B). This experimental time point was the first to result in at least some embryos without phenotypic defects, suggesting that 8 h of β-catenin production following fertilization is approaching the endpoint for the requirement of nuclear β-catenin during early *P*. *lividus* development. In support of this statement, we found that blocking β-catenin translation starting at 10 hpf and 12 hpf (late and swimming blastula stage, respectively) resulted in embryos identical to those obtained from β-cateninM+β-cateninPM-injected embryos that were not exposed to UV light. Indeed, upon UV exposure at 10 hpf and 12 hpf we obtained, respectively, 71% (39/55) and 70% (48/69) of normal late gastrulae and 29% (16/55) and 30% (21/69) of delayed gastrulae (Fig 6B). Taken together, these morphological observations suggest that 20 min, 4 h, 6 h, and 8 h of β-catenin protein production upon fertilization are insufficient for enabling proper vegetal germ layer development and animal territory restriction during early *P*. *lividus* embryogenesis. However, if the embryo is provided with 10 h of β-catenin protein production following fertilization (and thus with at least 10 h of nuclear β-catenin activity), morphological development proceeds normally, at least until 24 hpf.

### Time point-specific blockage of β-catenin translation and development of the skeletogenic mesoderm

**Fig 7:**
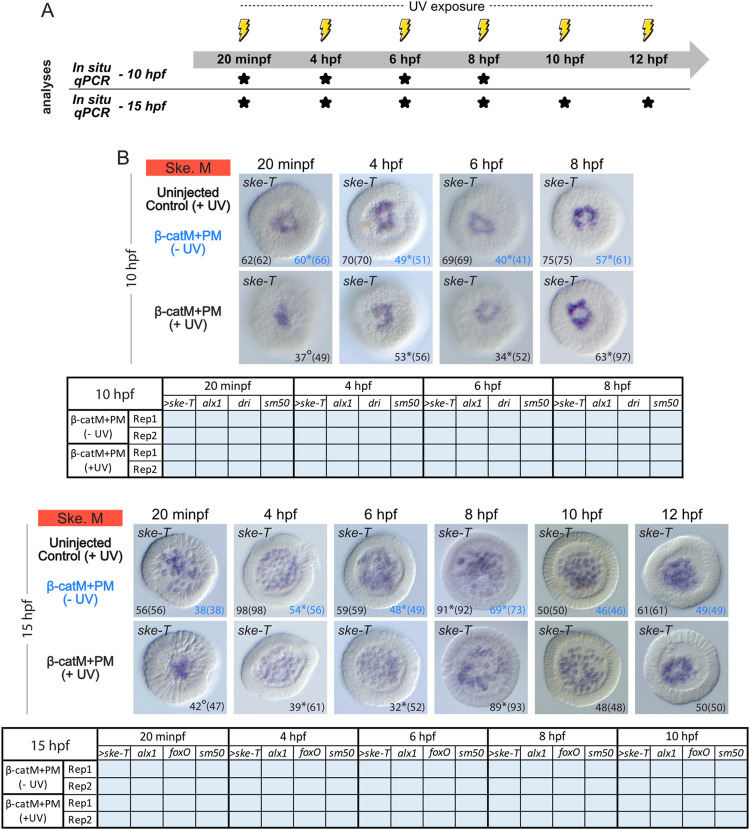
Time point-specific blockage of β-catenin translation and skeletogenic mesoderm development. (A) Schematic representation of the performed analyses, following the experimental procedure detailed in Fig 6A. For the time points marked by *, embryos were collected at 10 hpf (late blastula stage) and/or 15 hpf (mesenchyme blastula stage) [37] to carry out *in situ* hybridization (*In situ*) and quantitative RT-PCR (qPCR) assays. (B) *In situ* hybridization assays for the skeletogenic mesoderm (Ske. M) marker *ske-T* (*tbr*) [39] and qPCR expression matrices, from 2 independent biological replicates (Rep1 and Rep2), for *ske-T* and 4 additional skeletogenic mesoderm marker genes (*alx1*, *dri*, *foxO*, *sm50*). Experimental conditions included uninjected, irradiated control embryos: uninjected control (+UV); β-cateninM+β-cateninPM and not irradiated embryos: β-catM+PM (-UV); β-cateninM+β-cateninPM and irradiated embryos: β-catM+PM (+UV). The germ layer color-code is as in Fig 1. For the *in situ* hybridization assays, embryos are in vegetal view. Numbers in the bottom right and left corners indicate phenotypic counts relative to the total number of scored embryos (in parentheses). The 2 control conditions are shown in different. * and ° indicate, respectively, weak and no signal in the remaining scored embryos. For the qPCR analyses, results are shown as tables, and > marks *ske-T* that was also analyzed by *in situ* hybridization. Blue backgrounds highlight unaltered expression compared to uninjected, irradiated control embryos. Related raw data and quantification methods are available in S1 Data and fold change values are further provided in S2 Data.

We subsequently addressed the spatiotemporal requirements of nuclear β-catenin for the development of each of the vegetal germ layers, starting with the skeletogenic mesoderm (Fig 7). Using the experimental procedure detailed in Fig 6A, we carried out the analyses presented in Fig 7A. In controls (i.e., embryos uninjected and irradiated and embryos microinjected with β-cateninM+β-cateninPM but not irradiated), the majority of scored embryos were characterized by normal, unaltered expression profiles of the skeletogenic mesoderm marker *ske-T* (*tbr*) [38,39], and this no matter the time point of UV exposure and whether assessed at 10 hpf or 15 hpf (late and mesenchyme blastula stage, respectively) (Fig 7B). At 10 hpf, *ske-T* mRNA expression was detectable as a ring of epithelial cells surrounding the vegetal pole, and, at 15 hpf, *ske-T* mRNAs were found in scattered cells within the blastocoel, as the skeletogenic mesoderm underwent epithelial-to-mesenchymal transition.

In embryos microinjected with β-cateninM+β-cateninPM and exposed to UV light at 20 minpf, *ske-T* expression was also detectable, at both 10 hpf and 15 hpf, and this in a majority of scored embryos (75.5% (37/49) at 10 hpf and 89% (42/47) at 15 hpf). Accordingly, the expression levels of *ske-T* and of the 4 other skeletogenic mesoderm marker genes assayed by qPCR were unchanged in microinjected embryos exposed to UV light at 20 minpf, when compared to controls (Fig 7B). However, *in situ* hybridization assays revealed that, at both 10 hpf and 15 hpf, *ske-T* mRNAs were detected in fewer cells in β-cateninM+β-cateninPM embryos irradiated at 20 minpf than in controls. In addition, at 10 hpf, the expression of *ske-T* was patchy instead of forming a discrete ring (Fig 7B), and at 15 hpf *ske-T* remained in cells embedded in the epithelium rather than being scattered in the blastocoel (Fig 7B). Thus, although impairing β-catenin translation starting before fertilization abolishes expression of skeletogenic mesoderm genes (Fig 5B) allowing 20 min of β-catenin production upon fertilization is sufficient for initiating skeletogenic mesoderm fate specification (Fig 7B). However, this short pulse of β-catenin protein production with its associated nuclear β-catenin activity remains insufficient for properly instructing the complete development of this vegetal germ layer.

The later release of β-cateninM, at 4 hpf, 6 hpf, 8 hpf, 10 hpf, and 12 hpf (respectively, 16-cell, 60-cell, early blastula, late blastula, and swimming blastula stages), led to normal expression profiles of *ske-T* at both 10 hpf and 15 hpf in a majority of scored embryos in each experimental condition (at least 61.5% (32/52) obtained at 15 hpf following irradiation at 6 hpf) (Fig 7B). Likewise, equivalent results were obtained at 12 hpf following irradiation at 8 hpf (96% (82/85)) (S3 Fig). Under these experimental conditions, *ske-T* expression was always found in a total number of cells similar to that observed in controls and forming a ring at 10 hpf or being scattered in the blastocoel at 15 hpf (Fig 7B). Consistently, for each experimental condition, qPCR analyses yielded control-like expression levels for all assayed skeletogenic mesoderm markers (Fig 7C). Taken together, these observations support the notion that at most 4 h of β-catenin production following fertilization is sufficient in *P*. *lividus* not only for initiating, but also for completing the specification of the skeletogenic mesoderm, at least based on gene expression analysis and occurrence of epithelial-to-mesenchymal transition.

### Time point-specific blockage of β-catenin translation and development of the endomesoderm

**Fig 8:**
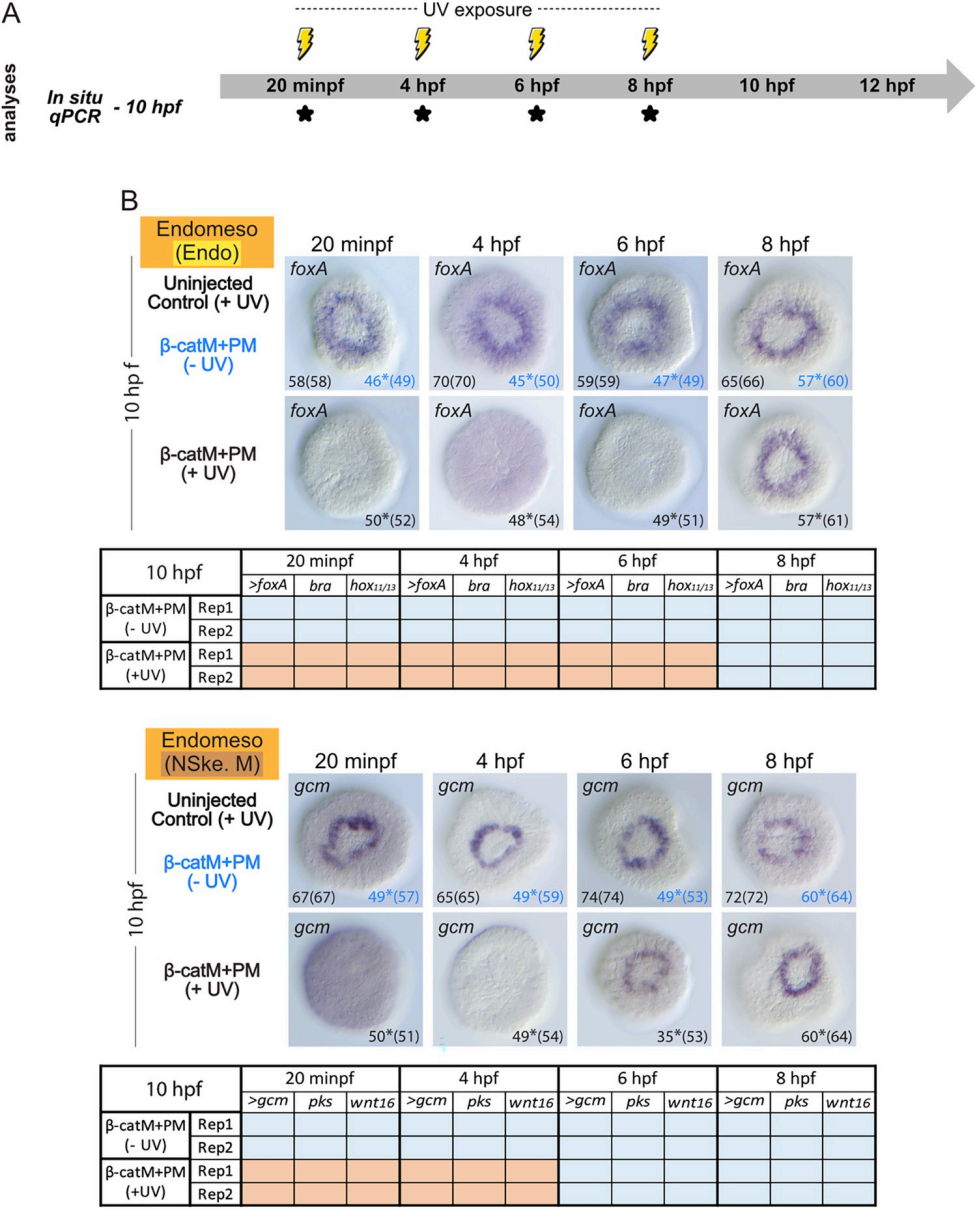
Time point-specific blockage of β-catenin translation and endomesoderm development. (A) Schematic representation of the performed analyses, following the experimental procedure detailed in Fig 6A. For the time points marked by *, embryos were collected at 10 hpf (late blastula stage) [37] to carry out *in situ* hybridization (*In situ*) and quantitative RT-PCR (qPCR) assays. (B) *In situ* hybridization assays for the 2 endomesoderm (Endomeso) marker genes *gcm* and *foxA* [26,28] and qPCR expression matrices, from 2 independent biological replicates (Rep1 and Rep2), for *gcm* and *foxA*, plus 4 additional co-expressed non-skeletogenic mesoderm (*pks*, *wnt16*) and endoderm (*bra*, *hox11/13*) marker genes. Experimental conditions included uninjected, irradiated control embryos: uninjected control (+UV); β-cateninM+β-cateninPM and not irradiated embryos: β-catM+PM (-UV); β-cateninM+β-cateninPM and irradiated embryos: β-catM+PM (+UV). The germ layer color-code is as in Fig 1. For the *in situ* hybridization assays, embryos are in vegetal view, and numbers in the bottom right and left corners indicate phenotypic counts relative to the total number of scored embryos (in parentheses). The 2 control conditions are shown in different colors, and * indicates weak signal in the remaining scored embryos. For the qPCR analyses, results are shown as tables, and > marks *foxA* and *gcm* that were also analyzed by *in situ* hybridization. Orange backgrounds indicate down-regulation by more than 2-fold compared to uninjected, irradiated control embryos, while blue backgrounds highlight unaltered expression compared to controls. Related raw data and quantification methods are available in S1 Data and fold change values are further provided in S2 Data.

Our observations demonstrate that the endomesoderm lineage is already present at 9 hpf (mid-blastula stage) and that it is then split into endoderm and non-skeletogenic mesoderm by 12 hpf to 12.5 hpf (swimming blastula stages) (Fig 2). Next, we thus focused the analysis of the spatiotemporal roles of β-catenin during *P*. *lividus* embryogenesis on endomesoderm development looking at embryos collected at 10 hpf (late blastula stage) (Fig 8A). In the vast majority of scored control embryos (i.e., uninjected, irradiated embryos and embryos injected with β-cateninM+β-cateninPM and not exposed to UV light), we found that the 2 endomesodermal markers *foxA* (an endoderm-related marker) and *gcm* (a non-skeletogenic mesoderm-related marker) were expressed, at 10 hpf, in a ring of cells surrounding the presumptive vegetal pole (Fig 8B), as previously observed (Fig 2) [26,28]. In contrast, in embryos microinjected with β-cateninM+β-cateninPM and exposed to UV light, at either 20 minpf or 4 hpf (16-cell stage), we observed no expression of *foxA* and *gcm*, at 10 hpf, in the majority of scored embryos (for *foxA*, 96% (50/52) at 20 minpf and 89% (48/54) at 4 hpf, for *gcm*, 98% (50/51) at 20 minpf and 91% (49/54) at 4 hpf) (Fig 8B). Consistently, the qPCR surveys carried out on endomesodermal markers, including *foxA* and *gcm*, revealed a significant down-regulation (more than 2-fold) of all assayed genes upon release of β-cateninM at 20 minpf and 4 hpf (Fig 8B). These results hence suggest that a sustained production of β-catenin protein is required, for at least 4 h following fertilization, to enable the proper specification of endomesoderm fate.

The release of β-cateninM at 6 hpf (60-cell stage) led, at 10 hpf, to a majority of embryos devoid of *foxA* mRNA (96% (49/51)) (Fig 8B), while 66% (35/53) of these embryos expressed *gcm* mRNAs (Fig 8B). Expression of *gcm* in these embryos was similar to that in controls, i.e., in a ring of cells surrounding the vegetal pole (Fig 8B). Accordingly, the qPCR analyses showed a significant down-regulation (more than 2-fold) of the expression levels of all tested endoderm marker genes in embryos microinjected with β-cateninM+β-cateninPM and exposed to UV light at 6 hpf, while expression of all assayed non-skeletogenic mesoderm marker genes was unaffected in these embryos (Fig 8B). Therefore, production of β-catenin proteins for 6 hpf is sufficient to trigger non-skeletogenic mesoderm gene expression in *P*. *lividus*. Yet, in the same endomesodermal cells, a longer β-catenin protein production (and thus nuclear β-catenin activity) is required for the initiation of endoderm specification.

In support of this conclusion, we found that UV exposure of β-cateninM+β-cateninPM-injected embryos at 8 hpf (early blastula stage) resulted in normal expression of both *foxA* and *gcm* in the vast majority of scored embryos at 10 hpf (for *foxA*, 93% (57/61) and, for *gcm*, 94% (60/64)) (Fig 8B). This was also the case in microinjected embryos irradiated at 8 hpf and collected at 12 hpf (normal expression detected in 88% (79/90) for *foxA* and in 87% (76/87) for *gcm*) (S3 Fig). The qPCR assays on endoderm and non-skeletogenic mesoderm markers further yielded similar results, with no statistically significant differences in expression levels between controls and experimental embryos exposed to UV light at 8 hpf (Fig 8B). Thus, although endomesodermal cells simultaneously express endoderm and non-skeletogenic mesoderm markers during early *P*. *lividus* development (i.e., between 9 hpf and 11.5 hpf) (Fig 2), our experiments demonstrate that triggering their expression requires different durations of β-catenin production following fertilization (i.e., at least 6 h for the non-skeletogenic mesoderm and 8 h for the endoderm).

### Time point-specific blockage of β-catenin translation and development of the endoderm and non-skeletogenic mesoderm

**Fig 9:**
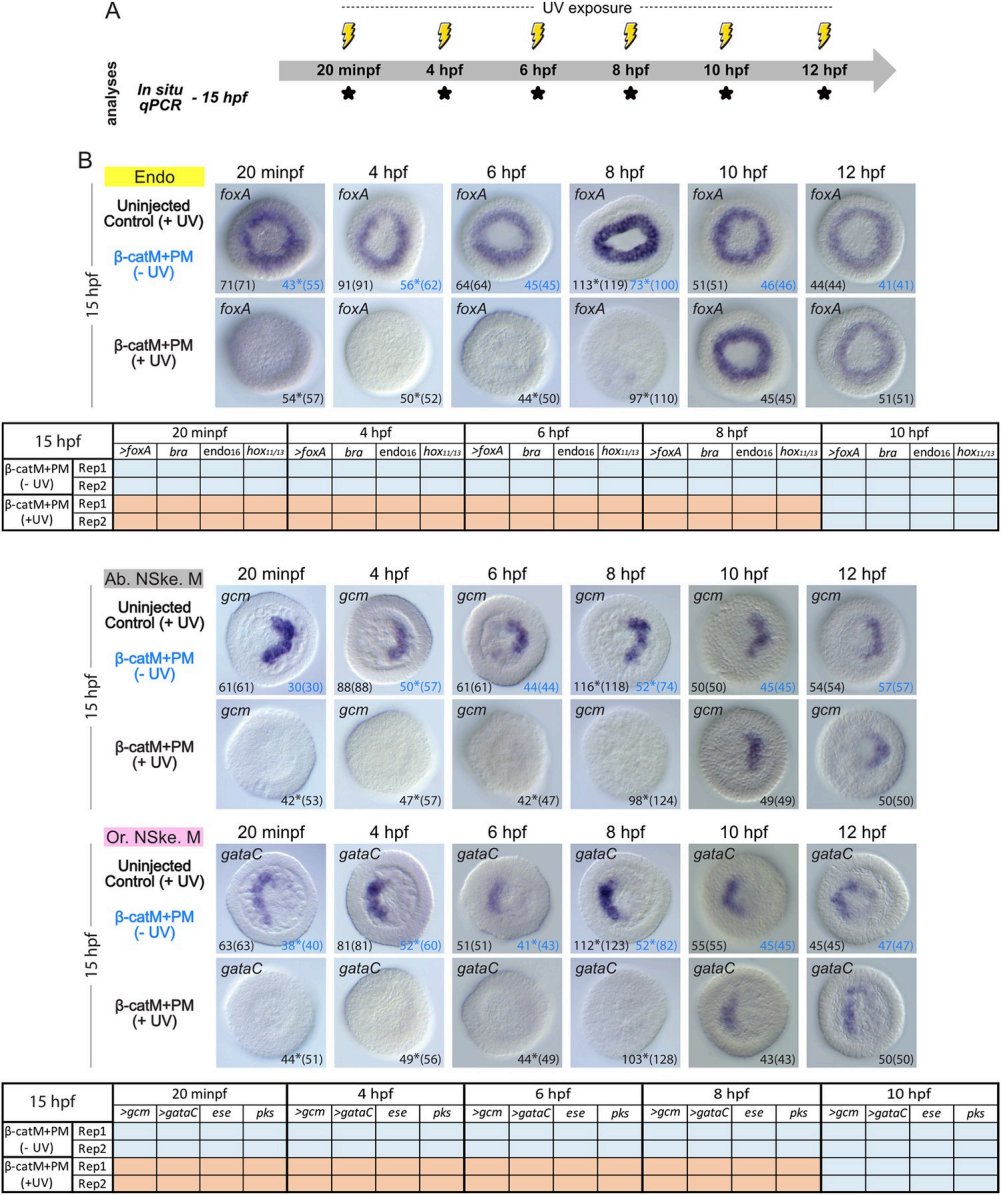
Time point-specific blockage of β-catenin translation and endoderm and non-skeletogenic mesoderm development. (A) Schematic representation of the performed analyses, following the experimental procedure detailed in Fig 6A. For the time points marked by *, embryos were collected at 15 hpf (mesenchyme blastula stage) [37] to carry out *in situ* hybridization (*In situ*) and quantitative RT-PCR (qPCR) assays. (B) *In situ* hybridization assays for the endoderm (Endo) marker gene *foxA*, the oral (ventral) non-skeletogenic mesoderm (Or. NSke. M) marker *gcm*, and the aboral (dorsal) non-skeletogenic mesoderm (Ab. NSke. M) marker *gataC* [26,48] and qPCR expression matrices, from 2 independent biological replicates (Rep1 and Rep2), for *foxA*, *gcm*, and *gataC* as well as 5 additional endoderm (*bra*, *endo16*, *hox11/13*) and oral (*pks*) and aboral (*ese*) non-skeletogenic mesoderm marker genes. Experimental conditions included uninjected, irradiated control embryos: uninjected control (+UV); β-cateninM+β-cateninPM and not irradiated embryos: β-catM+PM (-UV); β-cateninM+β-cateninPM and irradiated embryos: β-catM+PM (+UV). The germ layer color-code is as in Fig 1. For the *in situ* hybridization assays, embryos are in vegetal view. Numbers in the bottom right and left corners indicate phenotypic counts relative to the total number of scored embryos (in parentheses). The 2 control conditions are shown in different colors, and * indicates weak signal in the remaining scored embryos. For the qPCR analyses, results are shown as tables, and > marks *gcm*, *gataC*, and *foxA* that were also analyzed by *in situ* hybridization. Orange backgrounds indicate down-regulation by more than 2-fold compared to uninjected, irradiated control embryos, while blue backgrounds highlight unaltered expression compared to controls. Related raw data and quantification methods are available in S1 Data and fold change values are further provided in S2 Data.

We further addressed the spatiotemporal requirements of nuclear β-catenin during development of the endoderm and non-skeletogenic mesoderm lineages following their segregation from the endomesoderm (Fig 9). For this analysis, we focused on embryos collected at 15 hpf (onset of gastrulation, mesenchyme blastula stage) (Fig 9A), when the endoderm and non-skeletogenic mesoderm germ layers are segregated and characterized by the mutually exclusive expression of distinctive sets of marker genes (Fig 1) [26,28]. At 15 hpf, the non-skeletogenic mesoderm is further subdivided into an oral (ventral) and an aboral (dorsal) territory, each of which is defined by the expression of specific cohorts of genes (Fig 1) [48,49]. In the vast majority of scored controls (i.e., uninjected, irradiated embryos and β-cateninM+β-cateninPM-injected, not irradiated embryos), we found that, at 15 hpf, the endoderm marker *foxA* was expressed in a ring of cells surrounding the vegetal pole (Fig 9B), as previously described [50]. We also found that *gcm* and *gataC*, respectively marking the oral and aboral non-skeletogenic mesoderm, were expressed around the vegetal pole in 2 distinct, opposing half rings (Fig 9B), as expected [48]. *Gcm* mRNAs were in the aboral half ring, while *gataC* mRNAs were in the oral half ring (Fig 9B).

Following UV treatment at 20 minpf or 4 hpf (16-cell stage) of the β-cateninM+β-cateninPM-injected embryos, we failed to detect the expression of *foxA*, *gcm*, and *gataC*, at 15 hpf, in the vast majority of scored embryos (for *foxA*, 95% (54/57) at 20 minpf and 96% (50/52) at 4 hpf, for *gcm*, 79% (42/53) at 20 minpf and 82% (47/57) at 4 hpf, and, for *gataC*, 86% (44/51) at 20 minpf and 87.5% (49/56) at 4 hpf) (Fig 9B). Accordingly, the qPCR assays revealed a significant down-regulation (more than 2-fold), in these embryos, of the expression of all assayed endoderm and non-skeletogenic mesoderm markers (Fig 9C). These results are thus consistent with the previously determined 4 h requirement of β-catenin protein production after fertilization to trigger endoderm and non-skeletogenic mesoderm specification in the *P*. *lividus* embryo (Fig 8).

Following the release of β-cateninM at 6 hpf (60-cell stage) and 8 hpf (early blastula stage), we did not detect any expression of *foxA*, *gcm*, and *gataC* in most irradiated β-cateninM+β-cateninPM embryos at 15 hpf (for *foxA*, 88% (44/50) at 6 hpf and 88% (97/110) at 8 hpf, for *gcm*, 89% (42/47) at 6 hpf and 79% (98/124) at 8 hpf, and, for *gataC*, 90% (44/49) at 6 hpf and 80% (103/128) at 8 hpf) (Fig 9B). The qPCR assays, at 15 hpf, further corroborated these findings, highlighting a significant down-regulation (more than 2-fold) of the expression levels of all tested endoderm and non-skeletogenic mesoderm markers under experimental conditions (Fig 9B). However, these results contrasted with our *in situ* hybridization and qPCR observations on β-cateninM+β-cateninPM embryos irradiated at 6 hpf or 8 hpf and collected at 10 hpf (Fig 8). UV exposure at 6 hpf led to a loss of *foxA* expression at 10 hpf (but not of *gcm*), while UV exposure at 8 hpf had no noticeable effect on both *foxA* and *gcm* expression when assessed at 10 hpf. Taken together, these results indicate that production of β-catenin protein is not only required for 6 h and 8 h following fertilization to respectively trigger non-skeletogenic mesoderm and endoderm specification, but that an extended production and nuclear activity of β-catenin proteins is further necessary subsequently to maintain these 2 cell fates during development.

Blocking β-catenin translation at 10 hpf and 12 hpf (late and swimming blastula stage, respectively) did not affect the expression of *foxA*, *gcm*, and *gataC* (Fig 9B). A normal expression profile was observed for each of these genes in all scored embryos (for *foxA*, 100% (45/45) at 10 hpf and 100% (51/51) at 12 hpf, for *gcm*, 100% (49/49) at 10 hpf and 100% (50/50) at 12 hpf, and, for *gataC*, 100% (43/43) at 10 hpf and 100% (50/50) at 12 hpf). Accordingly, the qPCR experiments, at 15 hpf, revealed unchanged expression levels between controls and experimental conditions for all tested endoderm and non-skeletogenic mesoderm markers (Fig 9B). Altogether, these experiments demonstrate that, while β-catenin protein production is initially required from fertilization to 6 hpf and 8 hpf respectively to initiate, within the endomesoderm lineage, the non-skeletogenic mesoderm and the endoderm GRNs, a longer production of β-catenin protein, of about 10 h, is then needed to sustain the activity of these GRNs and to hence ensure the maintenance of these 2 germ layers during *P*. *lividus* development.

### Time point-specific blockage of β-catenin translation and restriction of animal territories

**Fig 10:**
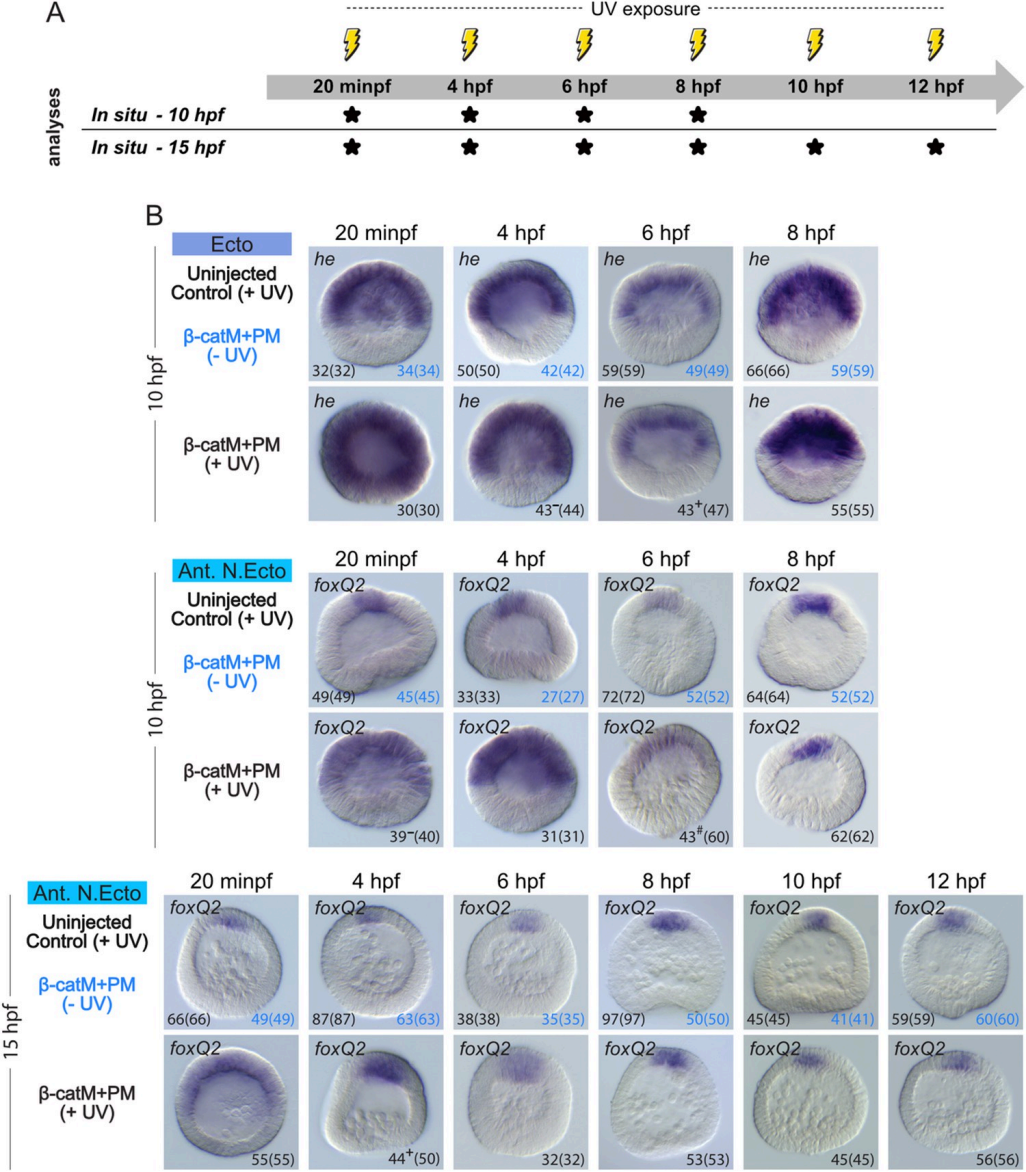
Time point-specific blockage of β-catenin translation and animal territory restriction. (A) Schematic representation of the performed analyses, following the experimental procedure detailed in Fig 6A. For the time points marked by *, embryos were collected at 10 hpf (late blastula stage) and/or 15 hpf (mesenchyme blastula stage) [37] to carry out *in situ* hybridization assays (*In situ*). (B) *In situ* hybridization assays for the ectoderm (Ecto) marker gene *he* and the anterior neuroectoderm (Ant. N.Ecto) marker *foxQ2* [21,54]. Experimental conditions included uninjected, irradiated control embryos: uninjected control (+UV); β-cateninM+β-cateninPM and not irradiated embryos: β-catM+PM (-UV); β-cateninM+β-cateninPM and irradiated embryos: β-catM+PM (+UV). The germ layer color-code is as in Fig 1, and embryos are in lateral view, with the animal pole up. Numbers in the bottom right and left corners indicate phenotypic counts relative to the total number of scored embryos (in parentheses). The 2 control conditions are shown in different colors. -, +, and # indicate that, relative to controls, the remaining scored embryos display, respectively, reduced, expanded, and unchanged expression domains.

Using our inducible, conditional knockdown approach, we also assessed the spatiotemporal requirements of nuclear β-catenin for restricting the ectoderm and anterior neuroectoderm territories along the animal-vegetal axis (Fig 10). For this analysis, we collected embryos at both 10 hpf (late blastula stage) and 15 hpf (onset of gastrulation, mesenchyme blastula stage) (Fig 10A). In controls (i.e., uninjected, irradiated embryos and embryos microinjected with β-cateninM+β-cateninPM and not irradiated), at 10 hpf, we detected expression of the ectoderm marker gene *hatching enzyme* (*he*) in the animal two-thirds of the embryo (Fig 10B) and that of the anterior neuroectoderm marker gene *foxQ2* in a more restricted animal territory corresponding to the animal pole domain (Fig 10B), as previously described [21,54]. Given that the expression of *he* is down-regulated at hatching (i.e., at 11 hpf) [55], we then only assessed, at 15 hpf, the expression of *foxQ2*, which, at this stage, persisted in the animal pole domain of all control embryos (Fig 10B), as previously reported [54].

In embryos microinjected with β-cateninM+β-cateninPM and exposed to UV light at 20 minpf, expression of both *he* and *foxQ2* was expanded at 10 hpf, when compared to controls (Fig 10B). This expansion was observed in 100% (30/30) of *he*-expressing and 97.5% (39/40) of *foxQ2*-expressing embryos. However, for both genes, this expansion was less severe than what was observed when β-cateninM was released before fertilization (compare Figs 5B and 10B). These results thus suggest that maintenance of β-catenin protein production, between fertilization and about 20 minpf, is necessary to contribute to the restriction of animal territories along the animal-vegetal axis in *P*. *lividus*. However, this production alone appears not to be sufficient for ensuring the complete restriction of the animal germ layers.

Release of β-cateninM at 4 hpf, 6 hpf, and 8 hpf (16-cell, 60-cell, and early blastula stages, respectively) led to progressively more restricted expression domains of *he* and *foxQ2* in embryos collected at 10 hpf (Fig 10B) (or at 12 hpf for the 8 hpf irradiation, S3 Fig). For *he* and *foxQ2*, control-like expression was obtained following a β-cateninM release at, respectively, 6 hpf and 8 hpf (for *he*, 91% (43/47) at 6 hpf and 100% (55/55) at 8 hpf, while, for *foxQ2*, 28% (17/60) at 6 hpf and 100% (62/62) at 8 hpf or if observed at 12 hpf 100% (46/46) for the 8 hpf irradiation) (Figs 10B and S3). These results allow 2 conclusions about the restriction of the ectoderm and anterior neuroectoderm territories along the animal-vegetal axis: (1) the restriction depends on a steady production and nuclear activity of β-catenin proteins starting at fertilization, to ensure its progressive continuity through development; and (2) the restriction is responsive to different β-catenin protein production periods following fertilization depending on the territory considered, i.e., one lasting for about 6 h for the ectoderm and one lasting for about 8 h for the anterior neuroectoderm.

At 15 hpf (mesenchyme blastula stage), we observed that the release of β-cateninM at 20 minpf led, in all scored embryos (100% (55/55)), to a distribution of *foxQ2* that was similar to that observed in embryos irradiated before fertilization and collected at 15 hpf (compare Figs 5B and 10B). Release of β-cateninM at 4 hpf and 6 hpf (16- and 60-cell stages, respectively), however, led to a more significant restriction of the *foxQ2* expression domain at 15 hpf (88% (44/50) at 4 hpf and 100% (32/32) at 6 hpf), when compared to β-cateninM release before fertilization (Fig 5B) or at 20 minpf (Fig 10B). Upon irradiation at 4 hpf and 6 hpf, expression of *foxQ2* was thus detectable in a small patch of cells at the animal pole, which was nonetheless about twice the size of that in controls (Fig 10B). It was only following the release of β-cateninM at 8 hpf, 10 hpf, and 12 hpf (early, late, and swimming blastula stages, respectively), that we found control-like expression of *foxQ2* at 15 hpf in all scored embryos (100% (53/53) at 8 hpf, 100% (45/45) at 10 hpf, and 100% (56/56) at 12 hpf) (Fig 10B). Taken together, these results indicate that restriction of the anterior neuroectoderm is dependent on at least 2 β-catenin activities: one early requiring protein production at fertilization and one late relying on protein production starting between 20 min and 4 h after fertilization.

## Discussion

**Fig 11:**
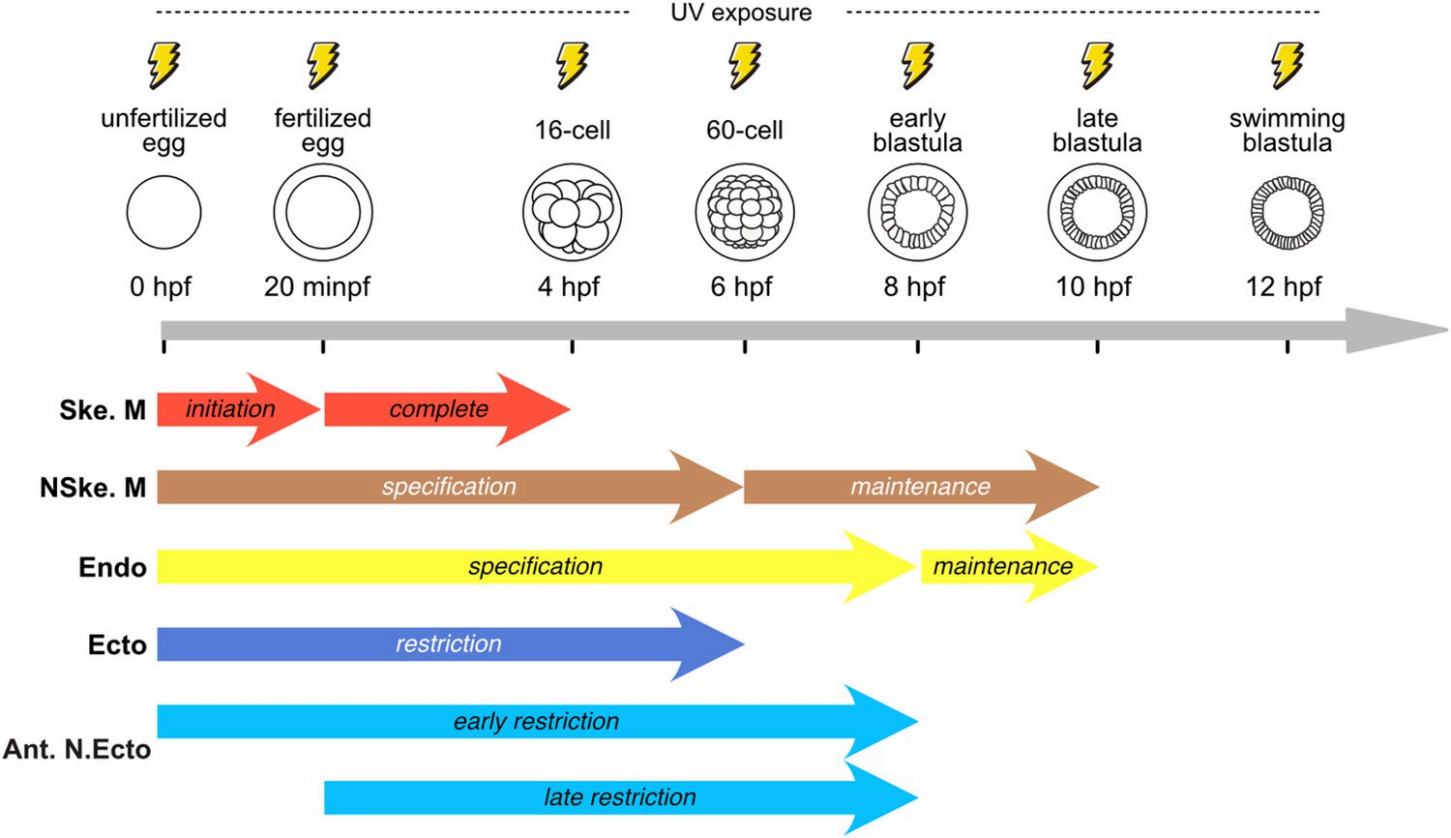
Schematic summary of the spatiotemporal requirements of nuclear β-catenin during early embryogenesis of the sea urchin *Paracentrotus lividus*. The top row illustrates the developmental stages and time points, in minpf and hpf, when conditional loss of β-catenin translation was induced by exposure to UV light. The bottom rows show the periods of β-catenin protein production required for the initiation and completion of skeletogenic mesoderm (Ske. M) development, for triggering and maintaining non-skeletogenic mesoderm (NSke. M) and endoderm (Endo) fates, and for restricting ectoderm (Ecto) and anterior neuroectoderm (Ant. N.Ecto) territories along the animal-vegetal axis. The germ layer color-code is as in Fig 1.

In this study, we have developed an inducible, conditional knockdown approach and used it to perform a comprehensive functional characterization of the spatiotemporal roles of nuclear β-catenin during early sea urchin development (Fig 11). Our analyses indicate that, following fertilization, different durations of β-catenin protein production (and thus of nuclear β-catenin activity) are required: (1) to trigger the specification of the 3 vegetal germ layers (skeletogenic mesoderm, non-skeletogenic mesoderm, and endoderm); (2) to subsequently maintain the specification of 2 of these germ layers (non-skeletogenic mesoderm and endoderm); and (3) to restrict the animal territories (ectoderm and anterior neuroectoderm) along the animal-vegetal axis. Our results further suggest that these duration requirements are correlated with the position of the targeted lineage along the animal-vegetal axis: (1) of the 3 vegetal germ layers, the most vegetal one, the skeletogenic mesoderm, needs the shortest β-catenin protein production period after fertilization for its proper development; and (2) of the 2 animal territories, the most animal one, the anterior neuroectoderm, requires a longer duration of β-catenin protein production following fertilization for its proper restriction than the ectoderm. In the following, we will discuss the tissue-specific β-catenin protein production requirements we identified in *P*. *lividus*, chiefly in the context of early sea urchin and metazoan embryogenesis.

### Nuclear β-catenin and skeletogenic mesoderm fate

It has previously been proposed that the nuclear accumulation of β-catenin responsible for skeletogenic mesoderm development takes place in the micromeres at the late 16-/early 32-cell stage [56,57], when endogenous nuclear β-catenin proteins first become detectable by immunohistochemistry [6]. In addition, it has previously been shown that inhibition of β-catenin activity before or at the time of fertilization leads to embryos deprived of skeletogenic mesoderm [19,58]. Here, we demonstrate that impairment of β-catenin translation induced before fertilization indeed leads to a loss of expression of all the skeletogenic mesoderm genes we assayed by *in situ* hybridization and qPCR. However, we found that blocking β-catenin translation starting at 20 min after fertilization results in normal expression of all tested skeletogenic mesoderm markers, which included both specification and differentiation genes (Fig 1). This result suggests that the production of β-catenin proteins in the first 20 min following fertilization is sufficient for generating enough nuclear β-catenin activity for launching the skeletogenic mesoderm GRN (Fig 1) [59] and for triggering skeletogenic mesoderm specification. However, it still remains to be established how this early β-catenin production window relates to the previously described nuclearization of β-catenin proteins in the micromeres at the late 16-/early 32-cell stage and how it induces skeletogenic mesoderm specification. A first possibility is that β-catenin proteins produced at and right after fertilization are stabilized and/or protected from the destruction complex until at least the late 16-cell stage. Although this hypothesis is supported by the enrichment, at the vegetal pole, of Dishevelled proteins [60], which enhance β-catenin accumulation in the nucleus [61], it is not consistent with the report that the destruction complex uniformly triggers β-catenin degradation in all blastomeres prior to the 16-cell stage [19]. A second possibility can be inferred from work carried out in frog embryos and human cells, which suggests that some developmental processes are activated by fold change increases of β-catenin protein level rather than by its overall concentration [62,63]. A simple fold change of β-catenin proteins might hence be sufficient in some contexts to initiate transcriptional regulation. Given that fertilization in *P*. *lividus* is accompanied by a burst of β-catenin protein synthesis [15], it is possible that the initiation of the skeletogenic mesoderm GRN in sea urchins relies also on such a fold change mechanism. In this case, induction of transcription might even occur before the 16-cell stage and rely on amounts of β-catenin protein that are below the detection limits of currently available imaging techniques.

When blocking β-catenin translation starting at 20 minpf, we further obtained embryos that expressed late components of the skeletogenic mesoderm GRN, such as *deadringer* and *sm50* (Fig 1), but without the skeletogenic mesoderm undergoing epithelial-to-mesenchymal transition. We retrieved embryos with skeletogenic mesoderm cells scattered within the blastocoel only when β-catenin translation was blocked starting at 4 hpf. These results suggest that, although production of β-catenin proteins for about 20 min following fertilization is sufficient for initiating the skeletogenic mesoderm GRN, a longer production for up to 4 h is necessary for ensuring the activation of the entire network, including the subset driving ingression of skeletogenic mesoderm cells into the blastocoel. In sea urchins, the cells establishing the skeletogenic mesoderm descend from the large micromeres [34,64], which first emerge at the 32-cell stage, corresponding to 5 hpf in *P*. *lividus* [37]. It is also known that, when sea urchin micromeres are isolated at the 16-cell stage (corresponding to 4 hpf in *P*. *lividus*) [37], they are already capable of producing large descendants and generate skeleton [65]. Our results are thus consistent with the notion that, by the 16-cell stage, the sea urchin embryo has already produced sufficient amounts of β-catenin proteins for the micromere descendants to be committed to the skeletogenic mesoderm fate and to subsequently undergo epithelial-to-mesenchymal transition. However, the exact timing of activation of genes within the skeletogenic mesoderm GRN remains to be established.

### Nuclear β-catenin and endomesoderm fate

Previous work carried out in different animal models has already demonstrated the requirement of nuclear β-catenin for the ontogeny of the endomesoderm (e.g., [66–68]). Here, in *P*. *lividus*, we corroborated that this requirement for β-catenin nuclearization starts at fertilization, as it has previously been shown, for example, in the sea urchin *S*. *purpuratus* [28], the cnidarian *Nematostella vectensis* [69], and the hemichordate *Saccoglossus kowalevskii* [70]. In S. *kowalevskii*, a sustained activity of nuclear β-catenin from fertilization to 10 hpf (the 256-cell stage) has further been reported as critical for endomesoderm specification [70]. Our results in *P*. *lividus* are consistent with this notion. However, we also established that different durations of β-catenin protein production after fertilization are needed within the endomesoderm to activate expression of non-skeletogenic mesoderm and endoderm genes (i.e., until, respectively, 6 hpf and 8 hpf). In the ascidian tunicate *Ciona intestinalis*, a sustained ON-ON sequence of nuclear β-catenin has been shown to be necessary for endoderm development, while an ON-OFF sequence is required for mesoderm development [12]. The segregation of the endomesoderm into endoderm and mesoderm in *C*. *intestinalis* is thus marked by the loss of β-catenin nuclearization in the mesoderm. In sea urchins, in contrast, segregation of the endomesoderm into endoderm and non-skeletogenic mesoderm takes place prior to the clearance of nuclear β-catenin from the vegetal cells, whether in *P*. *lividus* or in *L*. *variegatus* (this study, [26]). This suggests that, even though a different molecular mechanism is likely underlying the segregation of the endomesoderm lineage in ascidians and sea urchins, a longer activity or protein production of β-catenin is similarly required in both animals for the proper development of the endoderm. In mouse embryonic stem cells, establishment of the endoderm fate also requires a sustained activity of β-catenin [71].

Our double *in situ* hybridization assays in *P*. *lividus* further established that the split of the endomesoderm into endoderm and non-skeletogenic mesoderm is a gradual process during sea urchin development. Single-cell RNAseq data from *L*. *variegatus* have confirmed this observation in another sea urchin species [35]. Likewise, in human embryonic stem cells, gradual shifts of gene expression modules have been reported as the endoderm and the mesoderm segregate from a common endomesoderm lineage [72,73]. Moreover, previous work in *L*. *variegatus* has highlighted that the segregation of the endomesoderm into endoderm and non-skeletogenic mesoderm correlates with the down-regulation of *foxA* and the up-regulation of *delta* in non-skeletogenic mesoderm cells [26], an event that we also observed in *P*. *lividus*. Whether these 2 transcriptional events are functionally linked however remains elusive. Experiments carried out in *S*. *purpuratus* have indicated that silencing of *foxA* transcription in non-skeletogenic mesoderm cells is linked to TCF/LEF binding sites present in the promotor region of that gene [74]. From there, it has been suggested that the loss of *foxA* transcription in these cells was due to the clearance of nuclear β-catenin in the mesoderm cells, at the onset of gastrulation, as reported in *L*. *variegatus* [74]. However, as mentioned above, segregation of the endomesoderm into endoderm and non-skeletogenic mesoderm takes place, in *P. lividus* as in *L*. *variegatus*, prior to any clearance of nuclear β-catenin in vegetal cells [6,26]. In fish and mice, nuclear β-catenin has been shown to fulfill a dual function in the mesoderm, depending on the developmental stage considered [75,76]. In fish, for example, nuclear β-catenin promotes cardiac differentiation prior to gastrulation and inhibits heart formation during gastrulation [76]. It might thus be that during ontogeny of the sea urchin endomesoderm, like in vertebrates, nuclear β-catenin switches roles, at least with respect to the regulation of *foxA* transcription.

### Nuclear β-catenin and endoderm and non-skeletogenic mesoderm fates

In sea urchins, the endoderm and the non-skeletogenic mesoderm share several features: they both arise from the macromeres [64], their respective GRNs both require an early input of nuclear β-catenin [19], and they both co-express several marker genes before segregating into 2 distinct developmental territories [26,28]. Here, we established that both the endoderm and the non-skeletogenic mesoderm further require an equivalent sustained period of β-catenin production and activity, of up to 10 h following fertilization, for the maintenance of their fates through development. A similar requirement of nuclear β-catenin for an initial specification and a subsequent maintenance of a cell fate has previously been reported in primitive blood cells of frogs [77] and in neuromesodermal precursors of mice [78]. Despite these similarities, we also showed here that the transcriptional activation of endoderm and non-skeletogenic mesoderm marker genes is dependent on different durations of β-catenin protein production. In sea urchins, it is known that the development of non-skeletogenic mesoderm identity depends on the activity of both nuclear β-catenin and the Delta/Notch pathway (Fig 1). While nuclear β-catenin is initially required in the micromeres to trigger expression of Delta, the ligand of the Notch pathway, it is then Delta/Notch signaling that drives non-skeletogenic mesoderm specification in juxtaposed cells [57,79]. Subsequently, nuclear β-catenin is also needed in macromere descendants to ensure non-skeletogenic mesoderm development [56], even though no nuclear β-catenin target genes have so far been identified in this germ layer [80,81]. This dual requirement of nuclear β-catenin in the non-skeletogenic mesoderm, and in particular its presence in the micromeres, is likely to at least partially explain the differential production requirements of β-catenin in the non-skeletogenic mesoderm and the endoderm. In support of this notion, previous work carried out in *P*. *lividus* on the function of the Wnt6-Frizzled1/2/7 ligand–receptor pair has demonstrated that the down-regulation of this pair specifically inhibits nuclear accumulation of β-catenin in the macromeres at the 32-cell stage, which subsequently impairs endoderm but not non-skeletogenic mesoderm development [18].

Another difference between the endoderm and the non-skeletogenic mesoderm fate has been identified in previous work carried out in *S*. *purpuratus*: while endoderm development relies on Wnt-Frizzled interactions at the cell membrane, the specification and subsequent differentiation of the non-skeletogenic mesoderm are independent of these interactions [16]. In this context, it has been proposed that the nuclear accumulation of β-catenin proteins, which is detectable in macromeres [6] and which is responsible for non-skeletogenic mesoderm development [56], solely relies on the enrichment, in the macromeres, of the cytoplasmic effector Dishevelled [60]. However, this conclusion is inconsistent with the reported role, in *P*. *lividus*, of the Wnt6-Frizzled1/2/7 ligand–receptor pair [18]. In light of these previous reports and our current results, we thus propose that in macromeres: (1) Dishevelled is responsible for a low, basal level of nuclear β-catenin accumulation, which is not detectable by immunohistochemistry but is sufficient for triggering development of the non-skeletogenic mesoderm; and (2) the Wnt6-Frizzled1/2/7 ligand–receptor pair acts in parallel to Dishevelled to increase the level of nuclear β-catenin, which is detectable by immunohistochemistry and is necessary for development of the endoderm. If correct, this scenario thus implies that, in addition to a difference in the required duration of β-catenin production, the ontogeny of the endoderm and non-skeletogenic mesoderm lineages further relies on different doses of nuclear β-catenin activity, a notion that is reminiscent of the situation in the sea star *P*. *miniata* [14].

### Nuclear β-catenin and ectoderm and anterior neuroectoderm fates

In sea urchins, restriction of the ectoderm and anterior neuroectoderm territories along the animal-vegetal axis is regulated by an interplay of several Wnt signaling components, including nuclear β-catenin, JNK, and PKC as well as Wnt receptor antagonists, such as Dkk and sFRP [21,82]. Restriction of the anterior neuroectoderm has been proposed to take place in several steps, with nuclear β-catenin being required between the 16- and 32-cell stages [82]. Here, through the use of our inducible knockdown approach, we showed that an early accumulation of nuclear β-catenin is indeed required for restricting not only the anterior neuroectoderm but also the ectoderm. We found, however, that this accumulation has to take place prior to the 16-cell stage, starting at fertilization, and that a sustained production of β-catenin proteins is also necessary thereafter, until at least 6 and 8 h after fertilization, to progressively restrict, respectively, the ectoderm and the anterior neuroectoderm along the animal-vegetal axis. We further identified a second window of β-catenin production, from about 20 minpf to 8 hpf, that is required for restricting the anterior neuroectoderm. How exactly this second production window of β-catenin is contributing to the Wnt pathway-dependent control of animal-vegetal axis patterning remains to be determined. Our results in *P*. *lividus* embryos at 15 hpf also indicate that this second β-catenin production window might mediate the restriction of the anterior neuroectoderm in a 2-step process. If confirmed, this would mean that a total of 3 β-catenin nuclearization events are involved in restricting the anterior neuroectoderm during sea urchin embryogenesis, a hypothesis that will have to be tested in future analyses.

In this work, we also determined that restriction of the anterior neuroectoderm relies on the action of at least one nuclear β-catenin-independent mechanism. Based on the multi-step model proposed for restriction of the sea urchin anterior neuroectoderm [82], one possible candidate for this mechanism is the Frizzled/JNK pathway. However, the initial activation of this pathway has been proposed to be driven by nuclear β-catenin at the vegetal pole [82]. In vertebrates, FGF and retinoic acid signaling have been shown to promote posterior, nuclear β-catenin-positive tissues and to inhibit anterior gene expression [83,84], with the FGF pathway acting independently of a Wnt signal [85]. While thorough analyses of retinoic acid signaling functions during early sea urchin development remain elusive, previous work on the FGF receptors FGFR1 and FGFR2 in *P*. *lividus* has established that at least *fgfr1* is abundantly expressed in the ectoderm and anterior neuroectoderm in blastulae and that its expression is restricted to the anterior neuroectoderm at the onset of gastrulation [86]. However, down-regulation of FGFR1 and FGFR2 translation has been reported not to affect gene expression at the animal pole or restriction of ectoderm and anterior neuroectoderm territories [87].

## Conclusion

Using an innovative approach to induce gene knockdown at various stages during early sea urchin development, we characterized the spatiotemporal roles played by β-catenin nuclearization during germ layer specification, maintenance, and restriction (Fig 11). By blocking β-catenin protein production at different developmental stages, we identified novel developmental requirements for nuclear β-catenin and provided new mechanistic insights into the commitment of endomesoderm fate and into the restriction of animal territories. Our findings in the sea urchin are reminiscent of nuclear β-catenin functions in cnidarians, annelids, hemichordates, ascidian tunicates, and vertebrates (e.g., [13,44,45,47,48,64]). Taken together, this work nicely complements our current knowledge on the roles of nuclear β-catenin in developing sea urchins and adds substantial new experimental evidence for a better understanding of the dynamics of β-catenin functions during early animal development.

## Materials and methods

### Adults and embryos

*P*. *lividus* adults were collected in the bay of Villefranche-sur-Mer, France. Gametes were obtained as previously described [55], and embryos were cultured at 18°C in either artificial seawater (ASW) (516 mM NaCl, 11 mM CaCl_2_, 10 mM KCl, 34 mM MgCl_2_, 22 mM MgSO_4_, 10 mM Tris-HCl (pH 8.0), adjusted to pH 8.2) or Millipore-filtered seawater (MFSW) (0.2 μm). Embryo staging was carried out according to the developmental atlas of *P*. *lividus* [37].

### Embryo treatments and handling

Treatments with lithium chloride (LiCl) (#L9650, Sigma-Aldrich, Saint-Quentin-Fallavier, France) were carried out following standard procedures [88,89] and using a final concentration of 25 mM diluted in ASW. Treatments with UV light were performed as global irradiations of *in toto* embryos using an HBO 50W/AC L1 bulb mounted in a dark chamber. UV treatments were carried out at seven developmental stages: (1) before fertilization, before the establishment of any particular germ layer identity; (2) 20 minpf, just after the translational burst of β-catenin mRNA occurring at fertilization [15]; (3) 4 hpf (16-cell stage), just after the cell division giving rise to the micromeres and macromeres [64]; (4) 6 hpf (60-cell stage), after the emergence of the endomesoderm cells and the segregation of the skeletogenic mesoderm lineage [64]; and (5) 8 hpf; (6) 10 hpf; and (7) 12 hpf (early, late, and swimming blastula stage, respectively), before and after the endomesoderm lineage becomes detectable at the molecular level and once it segregates into endoderm and non-skeletogenic mesoderm (Figs 1 and 2). For UV treatments before fertilization, unfertilized, microinjected eggs were maintained in 10 ml ASW in 60 mm cell culture dishes. For UV treatments after fertilization, embryos were collected, upon fertilization, without their fertilization envelop, and placed in 4 ml ASW in 35 mm cell culture dishes. For all UV treatments, dishes were placed into the dark chamber at 12 cm from the HBO bulb, and irradiation was carried out at 60 mW/cm^2^ at room temperature (RT) for 20 min. Embryos were subsequently maintained in their respective cell culture dishes at 18°C until the desired developmental stages.

For morphology assessments upon UV treatments, embryos were collected at 24 hpf (late gastrula stage), when control embryos display a developing digestive tube (archenteron) in the blastocoel, along with well-organized skeletogenic mesoderm cells and scattered non-skeletogenic mesoderm cells. For *in situ* hybridization and qPCR assays, embryos were collected at 10 hpf (early blastula stage) and 15 hpf (mesenchyme blastula stage). At 10 hpf, the skeletogenic mesoderm is already segregated, but not the endoderm and the non-skeletogenic mesoderm (see Figs 1 and 2). At 15 hpf, all 3 vegetal germ layers are segregated (see Figs 1 and 2). For UV treatments at 8 hpf, *in situ* hybridization assays were also carried out on embryos collected at 12 hpf (swimming blastula stage) to ensure consistency of the results.

### β-cateninVenus (β-cateninV) cloning and imaging

To record β-catenin nuclear accumulation during *P*. *lividus* embryogenesis and to test our morpholino strategy, a β-catenin reporter construct, β-cateninV, was generated. The full-length open reading frame (ORF) of *P*. *lividus* β-catenin (823 amino acids) plus 55 base pairs upstream of its start codon (GenBank accession number: MT955895) were cloned into the expression vector pCS2+ [90] 5′ of the coding sequence of the Venus gene [91] (S1 Fig). The 55 base pairs upstream of the β-catenin ORF included the target site of the β-catenin antisense morpholino oligonucleotide (β-cateninM). To disable the regulatory activity of β-cateninV proteins, 2 mutations were introduced into the TCF/LEF binding domain of the β-catenin ORF by site-directed mutagenesis, changing the amino acids R487 and H488 to alanine (A) [92,93]. The distribution of β-cateninV proteins was assessed either in live embryos or by immunohistochemistry. When recorded live, embryos were mounted between a slide and a coverslip and imaged using a Zeiss Axio Imager 2 (A2) upright microscope (Zeiss, Jena, Germany) under differential interference contrast (DIC) light and epifluorescence. Images were taken using a 20× objective and were processed using either Adobe photoshop (version 12.0—CS5) (Adobe, San Jose, United States) or Affinity Photo (version 1.10.6) (Serif, Nottingham, United Kingdom). For immunohistochemistry, see details below.

### Morpholino oligonucleotides

Classical antisense (M) and photocleavable sense (PM) morpholino oligonucleotides specific for *P*. *lividus* β-catenin were designed and produced by Gene Tools, LLC (Philomath, Oregon, USA): β-cateninM (5′-TTCCAGTGAAATCTATCTAAGGGTC-3′) and β-cateninPM (5′-ACCCTTAGATAGATTTCACTGGAA-3′). Upon reception, each morpholino oligonucleotide was resuspended in RNase-free water to a final concentration of 3 mM, heated to 65°C for 10 min, aliquoted, and then stored at −80°C. Upon usage, morpholino oligonucleotide aliquots were thawed, heated to 65°C for 5 min, and diluted to their respective final concentrations.

### mRNA and morpholino oligonucleotide microinjections

All microinjections were performed into *P*. *lividus* oocytes prior to fertilization as previously described [21], and uninjected oocytes were used as controls. 5′ capped mRNAs encoding β-cateninV proteins were synthesized *in vitro* from linearized DNA using a mMessage mMachine kit (#AM1340, Fisher Scientific, Illkirch-Graffenstaden, France) and following the manufacturer’s instructions. 5′ capped mRNAs encoding the transmembrane plus intracellular domains of *L*. *variegatus* G-cadherin proteins were generated from the pBluescriptΔLvG-cadherin construct (ΔLvG-cadherin) (generously provided by David R. McClay), using the same procedure [6]. β-cateninV and ΔLvG-cadherin mRNAs were injected at final concentrations of, respectively, 0.2 μg/μl and 0.3 μg/μl prepared in RNase-free water. The β-cateninM and β-cateninPM morpholino oligonucleotides were respectively microinjected at 0.7 mM and 0.6 mM diluted in RNase-free water, and this when microinjected alone, together or with β-cateninV mRNAs.

### Single and double chromogenic *in situ* hybridization

GenBank accession numbers for the *in situ* hybridization probes used in this study are as follows: *delta*, DQ536193; *foxA*, ABX71819; *foxQ2*, MT955896; *gataC*, ACZ62636; *gcm*, ABG66953; *he*, X53598; *ske-T*, CAC51029. Single chromogenic whole-mount *in situ* hybridization experiments were performed as previously described [94], and each analysis was carried out in 3 independent biological replicates. Images were acquired using a Zeiss A2 upright microscope (Zeiss, Jena, Germany) under DIC light. Double chromogenic *in situ* hybridization for *delta* plus *gcm*, *delta* plus *foxA*, and *gcm* plus *foxA* were carried out using probes labeled with digoxigenin-11-UTP (DIG) (#11277073910 Roche, Meylan, France) and fluorescein-12-UTP (FLU) (#11685619910, Roche, Meylan, France). The protocol used was as previously described [94], except that the 2 probes were hybridized simultaneously at a final concentration of 1 ng/μl each. The FLU probes were developed first, using anti-FLU alkaline phosphatase conjugated antibodies (#11426338910, Roche, Meylan, France) and the Fast Red substrate (#F4648, Sigma-Aldrich, Saint-Quentin-Fallavier, France). The DIG probes were developed second, using anti-DIG alkaline phosphatase conjugated antibodies (#11093274910, Roche, Meylan, France) and the NBT (#11383213001, Roche, Meylan, France) plus BCIP (#11383221001, Roche, Meylan, France) substrates. Images were acquired using a Leica SP8 confocal microscope (Leica, Wetzlar, Germany). Excitations were at 543 nm for the Fast Red signal and at 633 nm for the NBT-BCIP signal. Two-channel acquisitions were performed sequentially. Channel 1 had a 565 nm to 585 nm band-pass filter for Fast Red detection, and channel 2 had a 700 nm to 800 nm band-pass filter for detection of the NBT-BCIP signal. For each sample, series of optical sections were taken at a z-step interval of 1 μm and confocal optical sections were subsequently compiled into maximum intensity z-projections using ImageJ (version 1.53c) [95]. Images were then processed using either Adobe Photoshop (version 12.0—CS5) (Adobe, San Jose, United States) or Affinity Photo (version 1.10.6) (Serif, Nottingham, United Kingdom).

### Immunohistochemistry

To record the temporal dynamics of β-cateninV proteins, a low dose of β-cateninV mRNAs (0.2 μg/μl) was used, compatible with the activity of the endogenous β-catenin destruction complex [17], and β-cateninV protein distribution was assayed by immunohistochemistry against the Venus protein. Immunohistochemistry for β-cateninV proteins was carried out using, as primary antibodies, polyclonal anti-GFP antibodies (#TP401, New England Biolabs, Evry, France). Embryos were fixed for 30 min at RT in 2% paraformaldehyde diluted in ASW containing 10 mM EPPS at pH 8.0. Following fixation, embryos were dehydrated for 2 min at RT in ice-cold methanol (MeOH). Embryos were then immediately rehydrated with 1 wash of 5 min at RT in 50% MeOH/50% 1× TBST (1× TBS, 0.05% Tween-20) and 1 wash of 5 min at RT in 1× TBST. Subsequently, embryos were washed once for 5 min at RT in 1× PBST (1× PBS, 0.05% Tween-20) and twice for 5 min each at RT in blocking solution (4% normal goat serum diluted in 1× PBS). Embryos were incubated overnight at 4°C with primary antibodies diluted at 1:200 in blocking solution. After 3 washes of 5 min each at RT in 1× PBST, embryos were incubated for 2 h at RT with secondary antibodies: goat anti-rabbit Alexa Fluor 488 antibodies (#A-11008, Thermo Fisher Scientific, Illkirch-Graffenstaden, France), diluted at 1:200 in blocking solution. Thereafter, 3 washes were carried out of 5 min each at RT in 1× PBST. Nuclear labeling was performed by incubation of the embryos for 5 min at RT in the dark in a solution of Hoechst 33342 (#B2261, Sigma-Aldrich, Saint-Quentin-Fallavier, France) diluted at 1 μg/ml in 1× PBST. 3 washes of 5 min each at RT were then performed in 1× PBST before mounting the embryos in 1× PBST. Images were acquired using a Leica SP8 confocal microscope (Leica, Wetzlar, Germany). Two-channel acquisitions were performed sequentially. For the GFP signal, the excitation was at 488 nm, and the band-pass filter was set from 506 nm to 551 nm. For the Hoechst signal, the excitation was at 405 nm, and the band-pass filter was set from 429 nm to 483 nm. For each sample, series of optical sections were acquired, and images were subsequently processed as described above for the double chromogenic *in situ* hybridization experiments.

### Co-*in situ* hybridization and immunohistochemistry

Co-*in situ* hybridization and immunohistochemistry labeling was carried out to assess, in the same embryos, the distribution of *foxA* mRNAs and β-cateninV proteins. Embryos were fixed for 30 min at RT in 2% paraformaldehyde diluted in ASW containing 10 mM EPPS at pH 8.0. For *foxA in situ* hybridization, we used a DIG-labeled (#11277073910, Roche, Meylan, France) probe and anti-DIG alkaline phosphatase conjugated antibodies (#11093274910, Roche, Meylan, France). For β-cateninV immunohistochemistry, we used polyclonal anti-GFP antibodies (#TP401, New England Biolabs, Evry, France) as primary antibodies and goat anti-rabbit Alexa Fluor 488 antibodies (#A-11008, Thermo Fisher Scientific, Illkirch-Graffenstaden, France) as secondary antibodies. The *in situ* hybridization assay was carried out as previously described [94] except that, after the blocking step, embryos were simultaneously exposed, for 2 h at RT, to the anti-DIG and the anti-GFP antibodies, respectively diluted at 1:2000 and 1:200 in blocking solution 1 (0.5% bovine serum albumin and 2% heat-inactivated goat serum diluted in 1× TBST). Embryos were then washed 3 times for 10 min each at RT in blocking solution 2 (4% normal goat serum diluted in 1× PBS), before being incubated for 2 h at RT with the secondary antibodies diluted at 1:200 in blocking solution 2. Subsequently, embryos were washed 6 times for 10 min each at RT in 1× TBST, before the *foxA in situ* hybridization signal was revealed using the NBT (#11383213001, Roche, Meylan, France) plus BCIP (#11383221001, Roche, Meylan, France) substrates. Images were acquired using a Leica SP8 confocal microscope (Leica, Wetzlar, Germany). The excitations used were at 488 nm for the Alexa Fluor 488 signal and at 633 nm for the NBT-BCIP signal. Two-channel acquisitions were performed sequentially. Channel 1 had a 495 nm to 530 nm band-pass filter for the Alexa Fluor detection, and channel 2 had a 700 nm to 800 nm band-pass filter for the detection of the NBT-BCIP signal. For each sample, series of optical sections were taken, and images were then processed as described above for the double chromogenic *in situ* hybridization experiments.

### Quantitative RT-PCR (qPCR)

The qPCR experiments were carried out as previously described [46,96]. Primers were designed from clone sequences available in the laboratory or from sequences retrieved from the transcriptome database available on the Octopus web portal (http://octopus.obs-vlfr.fr/). For the latter, sequences were retrieved by BLAST searches using as templates the annotated mRNA sequences from the sea urchin *S*. *purpuratus* available on EchinoBase (https://www.echinobase.org/echinobase/) and subsequently validated by reciprocal BLAST against the NCBI GenBank database (https://www.ncbi.nlm.nih.gov/nucleotide/). Primer sequences, sequence sources, and related GenBank accession numbers are provided in S4 Data. cDNA templates were generated from total RNA extracted from embryos obtained from 2 biological replicates for each experimental condition. For each cDNA preparation, 150 embryos were used, whether microinjected or not and UV-treated or not. Total RNA extraction was carried out using the RNeasy Mini Elute Cleanup kit (#74204, Qiagen, Les Ulis, France), and cDNA synthesis was performed using the SuperScript II Reverse Transcriptase kit (#18064022, Fisher Scientific, Illkirch-Graffenstaden, France), in both cases following the manufacturer’s instructions. qPCR reactions were performed in 96-well plates using the LightCycler 480 SYBR Green 1 master mix (#04707516001, Roche, Meylan, France) and the LightCycler 480 instrument (Roche, Meylan, France). Each qPCR reaction was carried out using 1.5 μl of cDNA in a final volume of 10 μl, and all qPCR experiments were performed in triplicates on the 2 independent cDNA preparations. The gene *β-tubulin* was used as an internal control to normalize gene expression levels, and fold changes were calculated using the 2^-ΔΔCt^ method [19] (raw data and quantification procedures are available in S1 Data). In brief, for data analysis, each cDNA preparation (i.e., biological replicate) was considered separately. Averages of control and experimental Ct values were calculated for each experimental qPCR triplicate series (AvgCt). The ΔCt value was then determined using ΔCt = AvgCt_gene_-AvgCt_β-tub_ and the ΔΔCt value using ΔΔCt = ΔCt_exp_-ΔCt_control_. Fold changes between experimental and control conditions were established using 2^-ΔΔCt^. The log_2_ (fold change) values for each gene and cDNA preparation are provided in S1 Data as well as reported in S2 Data.

## Supporting information

S1 Fig The β-cateninVenus (β-CateninV) construct and its *in vivo* validation. (A) Schematic representation of the β-cateninV construct, which includes: (1) the entire open reading frame (ORF) of the *Paracentrotus lividus* β-catenin protein, represented by gray and yellow boxes (including the putative phosphorylation sites, and the APC, TCF/LEF, and cadherin interaction domains); (2) 2 targeted mutations, R487 and H488 to alanine (A), to impair TCF/LEF binding, as previously demonstrated for human and ascidian β-catenin proteins [92,93]; (3) at the 5′ end, a 55-base pair domain corresponding to the 5′ UTR of the *P*. *lividus* β-catenin mRNA, which includes the β-catenin morpholino antisense oligonucleotide (MO) recognition site; and (4) at the 3′ end, the open reading frame encoding the Venus reporter protein. (B) First *in vivo* validation of the β-cateninV construct. The top row shows morphological phenotypes obtained at 72 h post fertilization (hpf) (4-arm pluteus stage), and the bottom row illustrates the distribution of β-cateninV proteins at 7 hpf (very early blastula stage), under the following 3 experimental conditions: (1) embryos microinjected with mRNA encoding β-cateninV and untreated (control); (2) embryos microinjected with mRNA encoding β-cateninV and treated with lithium chloride (LiCl) to up-regulate (+) the canonical Wnt/β-catenin signaling pathway [6,97]; and (3) embryos co-microinjected with mRNA encoding β-cateninV and a truncated form of cadherin (Δ-cad) to down-regulate (-) the canonical Wnt/β-catenin signaling pathway [6]. At 72 hpf, untreated control embryos developed into pluteus larvae, and, at 7 hpf, these embryos showed nuclear β-cateninV proteins only in the vegetal third. Embryos treated with LiCl were vegetalized and exogastrulated at 72 hpf, and, at 7hpf, they showed nuclear β-cateninV proteins in almost the entire embryo. Embryos co-microinjected with Δ-cad were animalized at 72 hpf, and, at 7 hpf, they were devoid of nuclear β-cateninV proteins. In these embryos, β-cateninV proteins were instead confined to cell membranes. Dotted lines on bottom row images highlight the area of the zoom shown in the lower left corner. (C) Second *in vivo* validation of the β-cateninV construct. Morphological phenotypes associated with the overexpression of the β-cateninV construct, microinjected as mRNA at different doses. At 24 hpf (late gastrula stage), no developmental defects were observed for any of the doses used. In (B, C), embryos are in lateral view with the animal or anterior pole up, and the ventral side to the right, when applicable.
(TIFF)

S2 Fig Clearance of β-cateninVenus (β-cateninV) proteins observed at the early mesenchyme blastula stage, i.e., at 14 h post fertilization (hpf). (A) Nuclear distribution of β-cateninV proteins as observed in live embryos collected and imaged at 14 hpf. (B) Maximum intensity projection of a confocal z-stack for an embryo collected at 14 hpf, fixed, and co-labeled by immunohistochemistry for β-cateninV proteins (green) plus DNA (blue).
(TIFF)

S3 Fig Additional molecular assays for the blocking of β-catenin translation at the early blastula stage, i.e., at 8 h post fertilization (hpf). Following UV treatment performed at 8 hpf, embryos were collected at 12 hpf (swimming blastula stage) to carry out *in situ* hybridization assays. Experimental conditions included uninjected, irradiated control embryos: uninjected control (+UV); β-cateninM+β-cateninPM and not irradiated embryos: β-catM+PM (-UV); β-cateninM+β-cateninPM and irradiated embryos: β-catM+PM (+UV). In (A–C), *in situ* hybridization assays for: (A) the skeletogenic mesoderm (Ske. M) marker gene *ske-T* [39]; (B) the 2 endomesoderm (Endomeso) marker genes, *gcm*, a non-skeletogenic mesoderm (NSke. M) marker, and, *foxA*, an endoderm (Endo) marker [26,28]; (C) the anterior neuroectoderm (Ant. N.Ecto) marker gene *foxQ2* [54]. In (A, B), embryos are in vegetal view. In (C), embryos are in lateral view, with the animal pole up. In (A–C), numbers in the bottom right and left corners indicate phenotypic counts relative to the total number of scored embryos (in parentheses). The 2 control conditions are shown in different colors. In (A, B), * indicates weak signal in the remaining scored embryos.
(TIFF)

S1 Data Raw data and quantification of fold changes upon β-cateninM release at different time points during *Paracentrotus lividus* embryogenesis. The document contains 4 sheets, respectively reporting the raw data of the qPCR results shown in Figs 5 and 7–9. The raw data are from 2 biological replicates (DNA sample—Rep) and 3 experimental replicates (R1-R3). Also included are the procedures for calculating the fold changes upon β-cateninM release compared to uninjected, irradiated control embryos and normalized relative to the expression of the reference gene *β-tubulin*.
(XLSX)

S2 Data Fold change values obtained upon β-cateninM release at different time points during *Paracentrotus lividus* embryogenesis. Fold change values are derived from the raw data and quantification procedures provided in S1 Data. The document contains 4 sheets. The first sheet displays the results obtained for all vegetal germ layer markers upon inhibition of β-catenin translation starting before fertilization (related to Fig 5). The following 3 sheets provide the results for inhibition of β-catenin translation at different time points after fertilization in a tissue-specific manner (related to Figs 7–9). In each sheet, only log2(2^-ΔΔCt^) values are provided. If the values are lower than the threshold of a 2-fold change, the cells are in orange. If the values are between 0- and 2-fold change (i.e., when expression levels are similar to those in uninjected, irradiated controls), the cells are in blue. In all sheets, > highlights genes for which *in situ* hybridization experiments have been carried out.
(XLSX)

S3 Data Raw counts and quantification of morphological phenotypes obtained upon β-cateninM release at different time points during *Paracentrotus lividus* embryogenesis. (XLSX)

S4 Data Primer sequences used for qPCR experiments. The primer sets were designed based on sequences from cloned genes (clone) or from transcriptome data (transcriptome). If from cloned genes, the corresponding GenBank accession number is provided in the table. If from transcriptome data, the sequences were retrieved by BLAST searches using as templates the annotated mRNA sequences from the sea urchin *Strongylocentrotus purpuratus* and subsequently validated by reciprocal BLAST against the NCBI GenBank database. The accession number of the corresponding NCBI sequence is provided in the table. Abbreviations: *P*. *lividus*, *Paracentrotus lividus*; *L*. *variegatus*, *Lytechinus variegatus*; *H*. *erythrogramma*, *Heliocidaris erythrogramma*.
(XLSX)

## Abbreviations

ASW: artificial seawater
DIC: differential; interference contrast
GRN: gene regulatory network
hpf: hours post fertilization
minpf: minutes post fertilization
MFSW: Millipore-filtered seawater
ORF: open reading frame
qPCR: quantitative RT-PCR
RT: room temperature
UV: ultraviolet
