# Supplementary figures and images for: Spatiotemporal requirements of nuclear β-catenin define early sea urchin embryogenesis
Source: PLoS Biol. 2024 Nov 12;22(11):e3002880. doi: 10.1371/journal.pbio.3002880 (PMC11661853; doi:10.1371/journal.pbio.3002880)

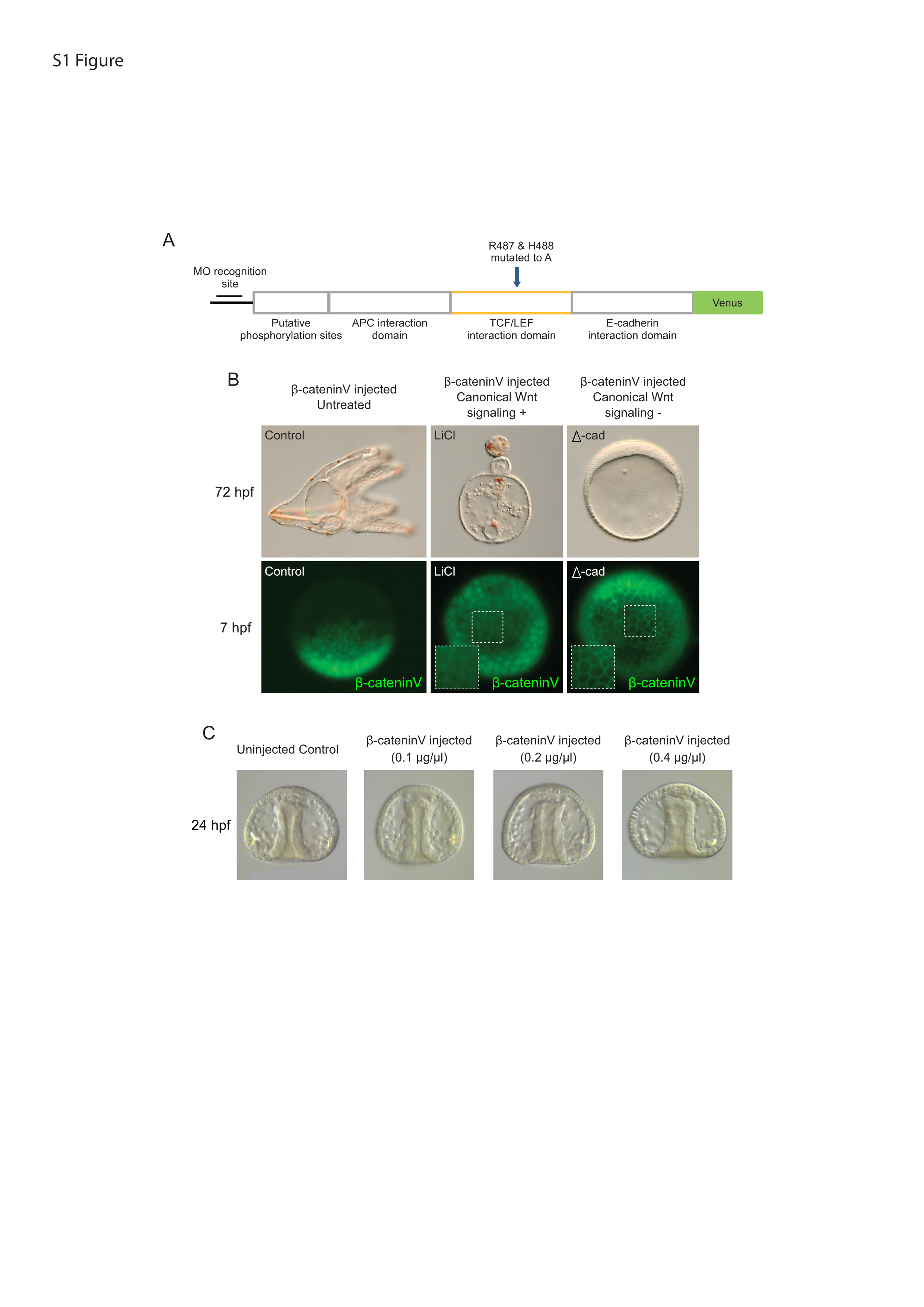

Supplement: S1 Fig — (A) Schematic representation of the β-cateninV construct, which includes: (1) the entire open reading frame (ORF) of the Paracentrotus lividus β-catenin protein, represented by gray and yellow boxes (including the putative phosphorylation sites, and the APC, TCF/LEF, and cadherin interaction domains); (2) 2 targeted mutations, R487 and H488 to alanine (A), to impair TCF/LEF binding, as previously demonstrated for human and ascidian β-catenin proteins [92,93]; (3) at the 5′ end, a 55-base pair domain corresponding to the 5′ UTR of the P. lividus β-catenin mRNA, which includes the β-catenin morpholino antisense oligonucleotide (MO) recognition site; and (4) at the 3′ end, the open reading frame encoding the Venus reporter protein. (B) First in vivo validation of the β-cateninV construct. The top row shows morphological phenotypes obtained at 72 h post fertilization (hpf) (4-arm pluteus stage), and the bottom row illustrates the distribution of β-cateninV proteins at 7 hpf (very early blastula stage), under the following 3 experimental conditions: (1) embryos microinjected with mRNA encoding β-cateninV and untreated (control); (2) embryos microinjected with mRNA encoding β-cateninV and treated with lithium chloride (LiCl) to up-regulate (+) the canonical Wnt/β-catenin signaling pathway [6,97]; and (3) embryos co-microinjected with mRNA encoding β-cateninV and a truncated form of cadherin (Δ-cad) to down-regulate (-) the canonical Wnt/β-catenin signaling pathway [6]. At 72 hpf, untreated control embryos developed into pluteus larvae, and, at 7 hpf, these embryos showed nuclear β-cateninV proteins only in the vegetal third. Embryos treated with LiCl were vegetalized and exogastrulated at 72 hpf, and, at 7hpf, they showed nuclear β-cateninV proteins in almost the entire embryo. Embryos co-microinjected with Δ-cad were animalized at 72 hpf, and, at 7 hpf, they were devoid of nuclear β-cateninV proteins. In these embryos, β-cateninV proteins were instead confined to cell me [file pbio.3002880.s001.tiff]

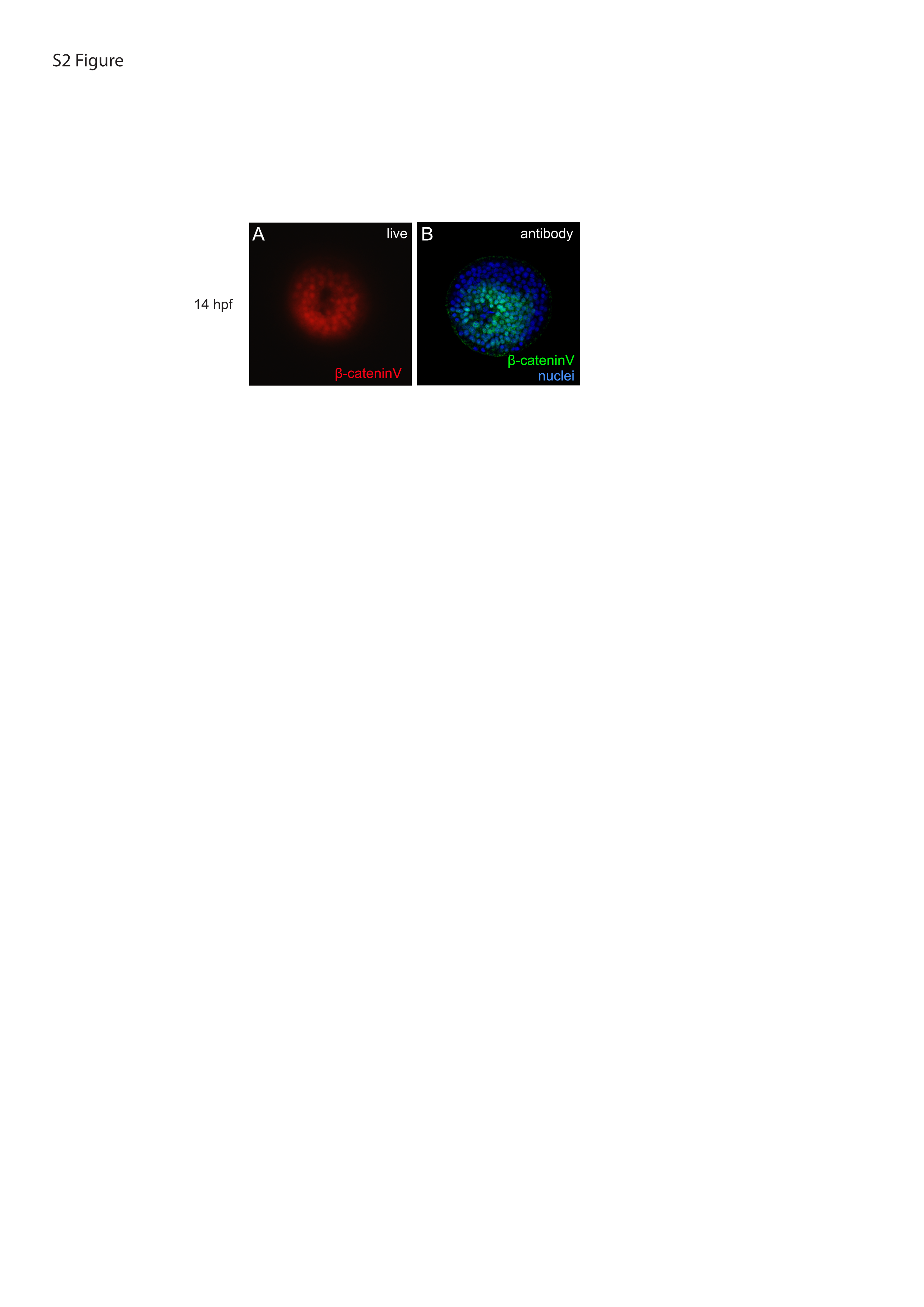

Supplement: S2 Fig — (A) Nuclear distribution of β-cateninV proteins as observed in live embryos collected and imaged at 14 hpf. (B) Maximum intensity projection of a confocal z-stack for an embryo collected at 14 hpf, fixed, and co-labeled by immunohistochemistry for β-cateninV proteins (green) plus DNA (blue). (TIFF) [file pbio.3002880.s002.tiff]

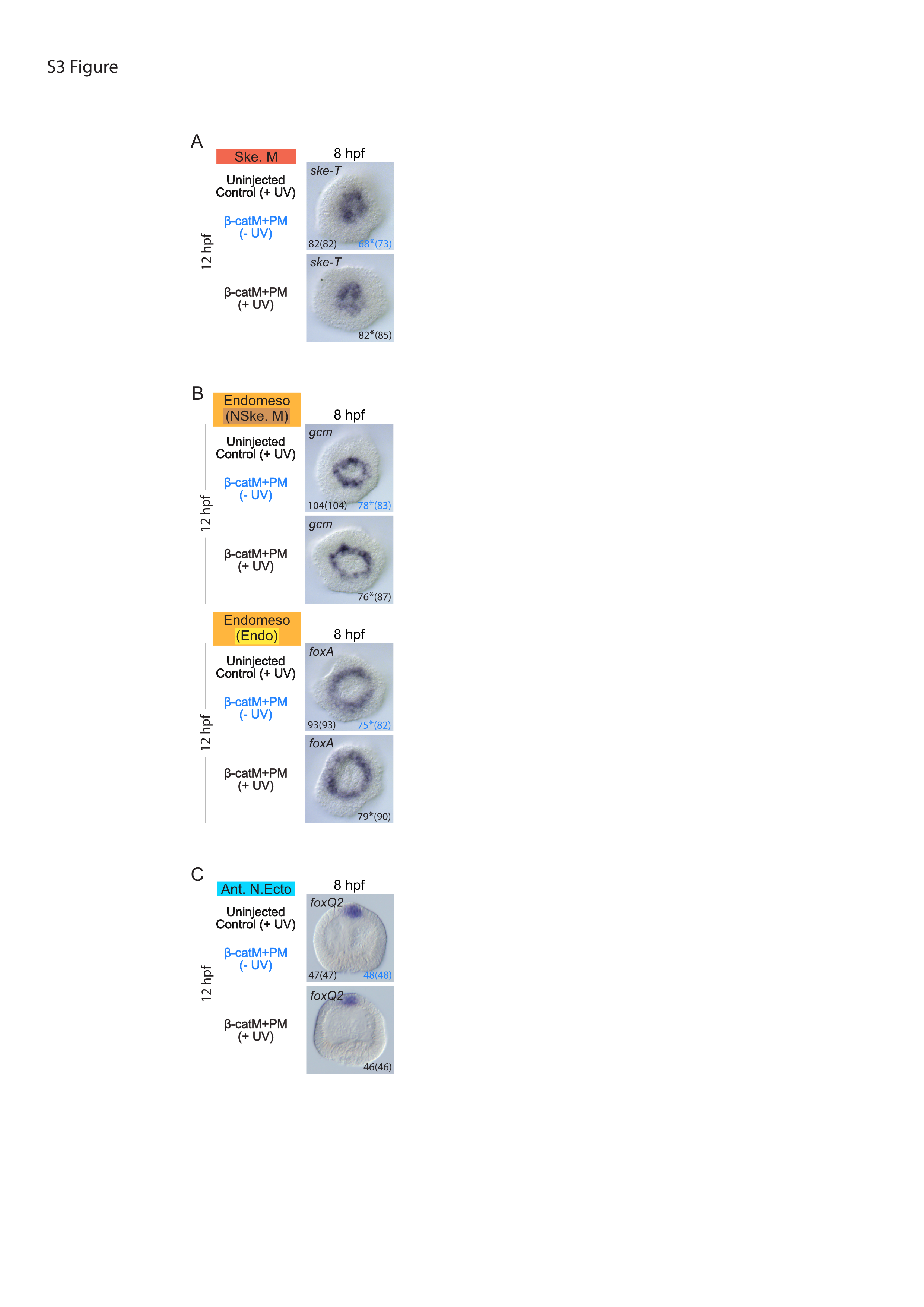

Supplement: S3 Fig — Following UV treatment performed at 8 hpf, embryos were collected at 12 hpf (swimming blastula stage) to carry out in situ hybridization assays. Experimental conditions included uninjected, irradiated control embryos: uninjected control (+UV); β-cateninM+β-cateninPM and not irradiated embryos: β-catM+PM (-UV); β-cateninM+β-cateninPM and irradiated embryos: β-catM+PM (+UV). In (A–C), in situ hybridization assays for: (A) the skeletogenic mesoderm (Ske. M) marker gene ske-T [39]; (B) the 2 endomesoderm (Endomeso) marker genes, gcm, a non-skeletogenic mesoderm (NSke. M) marker, and, foxA, an endoderm (Endo) marker [26,28]; (C) the anterior neuroectoderm (Ant. N.Ecto) marker gene foxQ2 [54]. In (A, B), embryos are in vegetal view. In (C), embryos are in lateral view, with the animal pole up. In (A–C), numbers in the bottom right and left corners indicate phenotypic counts relative to the total number of scored embryos (in parentheses). The 2 control conditions are shown in different colors. In (A, B), * indicates weak signal in the remaining scored embryos. (TIFF) [file pbio.3002880.s003.tiff]
